# Variability Assessment for Root and Drought Tolerance Traits and Genetic Diversity Analysis of Rice Germplasm using SSR Markers

**DOI:** 10.1038/s41598-019-52884-1

**Published:** 2019-11-11

**Authors:** H. Verma, J. L. Borah, R. N. Sarma

**Affiliations:** Department of Plant Breeding & Genetics, Assam Agricultural University, Jorhat, 785013 Assam India

**Keywords:** Agricultural genetics, Agricultural genetics, Plant breeding, Plant breeding

## Abstract

The studies on genetic variation, diversity and population structure of rice germplasm of North East India could be an important step for improvements of abiotic and biotic stress tolerance in rice. Genetic diversity and genetic relatedness among 114 rice genotypes of North East India were assessed using genotypic data of 65 SSR markers and phenotypic data. The phenotypic diversity analysis showed the considerable variation across genotypes for root, shoot and drought tolerance traits. The principal component analysis (PCA) revealed the fresh shoot weight, root volume, dry shoot weight, fresh root weight and drought score as a major contributor to diversity. Genotyping of 114 rice genotypes using 65 SSR markers detected 147 alleles with the average polymorphic information content (PIC) value of 0.51. Population structure analysis using the Bayesian clustering model approach, distance-based neighbor-joining cluster and principal coordinate analysis using genotypic data grouped the accession into three sub-populations. Population structure analysis revealed that rice accession was moderately structured based on F_ST_ value estimates. Analysis of molecular variance (AMOVA) and pairwise F_ST_ values showed significant differentiation among all the pairs of sub-population ranging from 0.152 to 0.222 suggesting that all the three subpopulations were significantly different from each other. AMOVA revealed that most of the variation in rice accession mainly occurred among individuals. The present study suggests that diverse germplasm of NE India could be used for the improvement of root and drought tolerance in rice breeding programmes.

## Introduction

Rice (*Oryza sativa* L.) is a primary staple food crop for more than 3.5 billion population of the world and cultivated across at least 114 countries^1^. Rice is grown on an area of 163 million hectares with the production of 758.9 million tonnes (499.2 million tonnes milled basis) of rice^2^. In India rice is cultivated on 43.79 million hectares with a production of 112.91 million tonnes of milled rice in 2017–18^3^. Rice production must be enhanced by about 60% to meet dietary needs by the year 2025 to match the explosive increase in world population^4^.

Rice is the main principal household cereal crop and nutritional security food crop of North East India. It occupies 75% of the total cultivated area of the region (4.58 million hectares)^5^. North-Eastern (NE) India being the secondary source centre of origin of rice, it is a hotspot of rice genetic resources in the world and rich in rice crop diversity. These landraces are grown in diverse ecosystems ranging from the high altitude of Arunachal Pradesh, the flood-prone areas of Assam, and rainfed, irrigated, upland, steep terraces and deep-water, *Jhum* and *tilla* land ecologies. Due to its wide spectra of cultivation systems and erratic rainfall due to climate change, rice cultivation in the NE region particularly Assam has experienced water stress condition in recent year’s drastically reducing yield. Now there has been tremendous pressure mounting on the breeders to identify the sources of resistance/tolerance to drought stress in rice to combat water stress condition.

Among the various groups of rice cultivated in Assam, upland rice cultivar of North East India which is directly sown in fields during March-April and harvested in July-August is known agronomically as “*aus/ahu”* rice^6^. These are photoperiod insensitive landraces maintained by farmers endowed with tremendous genetic variability and valuable genes for various abiotic stress tolerances as they are not subjected to selective breeding over a long period. Recently, with the use of genome sequence information, *aus* subpopulation is identified as distinct subpopulation from both *indica* and *japonica* subpopulation of *O. sativa* species^6–9^. Recent sequencing of wild rice relatives *Oryza rufipogon* species complex suggested that *aus* cultivars evolved from a distinct population of the annual *Oryza nivara* found in NE India, Bangladesh and Northern Myanmar^9,10^. Kim *et al*.^9^ stated that “the cultivated *aus* cultivars and its wild ancestor represent an underappreciated genetic resource”. These *aus* rice cultivars are early maturing, photoperiod insensitive and drought tolerant^8,11^. The genetic diversity among *aus* genotypes is abundant and they are enriched with various abiotic and biotic stress resistance genes^6,10,12,13^. For example, a traditional *aus* cultivar FR 13A harbour *Sub1* gene which confers submergence tolerance^14,15^; likewise Kasalath an *aus* type cultivar was the donor of *Pstol1* gene which confers phosphorus starvation tolerance^13^. Rayada, N-22 and Dular *aus* type cultivars have a large root length and high root density and drought resistance^16,17^. Nagina22 deep-rooted an *aus* type rice cultivar is the donor for heat and drought tolerant traits^18–23^. BG1222 an *aus* type cultivars harbour xa34 bacterial blight resistance genes^24^. Most of the drought-tolerant rice are originated from *aus* rice germplasm which has been cultivated in Northeast India and Bangladesh^12,25^. Knowledge of the genetic diversity of rice genotypes is useful for core collection development and effective conservation strategy. In the present study, an attempt was made to study genetic diversity in a few rice genotypes of North East India, the majority of which were agronomically identified as *aus/ahu* cultivars along with few *indica* rice.

The root system is the main part of plants for the absorption of water and nutrients from soil^26^. Root is the first organ to experience water stress and thus root system plays a vital role under drought stress conditions^27–29^. Root traits such as small fine root diameter, long specific root length and root length density play a major role in water uptake and maintaining plant productivity under drought. The nature and type of root characteristics are the main factors deciding their survival and adaptation to drought. The distribution of the root system and its density indicate water uptake potential^30^. In rice, deep rooting is governed by *DEEPER ROOTING1*, which confers improved drought resistance in drought stress environment^31^. In upland rice, the long root system is highly associated with drought tolerance^32^.

Study on root traits particularly in the field condition is very challenging and thus is very limited. Similarly, no attempt has been made to study the genetic architecture of the root traits in relation to drought tolerance using rice from North East India which necessitates the study of the diversity for such traits for supplement rice improvement works. It is envisaged that the selection of diverse parents based on root traits and drought tolerance would facilitate the development of transgressive segregates, as well as, heterotic groups for hybrid crop breeding in the population. However, genetic diversity analysis solely based on phenotypic traits may not be a reliable measure of genetic differences as they are influenced by environmental factors^33–35^. Thus, DNA based markers such as RAPD (Random Amplified Polymorphic DNA), SSR (Simple Sequence repeat), AFLP (Amplified Fragment Length Polymorphism) and SNP (Single Nucleotide Polymorphism) have been routinely used to assess the genetic divergence among the genotypes as they are not influenced by environmental factors.

Multi-allelic nature and high polymorphism of SSR markers help to establish the relationship among the individuals even with less number of markers^36^. SSR markers are preferred as they are abundance in the genome, well-distributed throughout the genome, hyper-variable, multi-allelic and co-dominant nature, ease of assaying, highly reproducible and highly informative^37,38^. SSR markers are immensely valuable in studies of variation detection, diversity analysis, phylogeny, population structure, gene mapping and association studies^39,40^.

The knowledge of the extent of genetic variation, diversity and genetic relationships between genotypes of the crop is vital and foundation for developing an improved cultivar possessing high yield, good grain quality and adapted to various abiotic and biotic stresses situations. Knowledge of the genetic diversity of rice genotypes is useful for core collection development and effective conservation strategy. Thus in this present study attempt has been made to (a) estimate the extent of genetic diversity in indigenous landraces using both morphological traits (drought tolerance, root and shoot traits) as well as SSR markers genotypic data. (b) To study the genetic structure in a few rice germplasm from NE India.

## Materials and Methods

### Plant materials

The experimental material for the present investigation comprised of 114 genotypes of rice (Table 1). Pure seeds of 114 genotypes were collected from Regional Agricultural Research Station (RARS), Titabar farm of Assam Agricultural University, Jorhat. These genotypes were directly sown in moist soil and evaluated in three replication for various root and shoot traits and drought tolerance using PVC pipes in a rain shelter at ICR (Instrumental cum Research) farm of Assam Agricultural University, Jorhat. Mean performance was recorded for root length, fresh root weight, dry root weight, root volume, root angle, bottom root number, peripheral root number, shoot length, dry shoot weight, fresh shoot weight, root to shoot ratio and ratio of deep rooting (Table 1). The root angle, bottom root number and peripheral root number were measured using basket method^41^. The root length, shoot length, fresh root weight and shoot weight data of 45-day-old plants were recorded using PVC pipes following standard method^42^. Plants were sampled and dried at 80 °C for seven days and root dry weight and shoot dry weight was recorded. The seedling stage drought tolerance score was recorded separately by growing the genotypes in the seedbed of three-row of 2-meter length per genotype in three replication under rain protected condition following Swain *et al*.^43^. The observation for drought tolerance was recorded at 32 and 34 days after withdrawal of life-saving irrigation (DAWW) when the soil moisture content was around 7–8% (W/V) and susceptible check (IR64 and Ranjit) showed complete drought stress symptoms using “Standard Evaluation System for Rice (SES)”^44^. The Soil moisture status was determined by the gravimetric method^45^.

**Table 1:** Mean performance of 114 genotypes for root, shoot and drought tolerance traits.

Sl. No.	Genotype	Drought Score 32 DAWI	Drought Score 34 DAWI	Recovery	Root Length (cm)	Shoot Length (cm)	Fresh Root Weight (gm)	Dry Root Weight (gm)	Fresh Shoot Weight (gm)	Dry Shoot Weight (gm)	Root Volume (ml)	Root Angle (0)	Periphery Root Number	Bottom Root Number	Root: Shoot Ratio	Ratio of Deep Rooting
1	AhuJoha	0.95	0.95	0.04	9.50	44.67	0.47	0.09	1.68	0.34	0.08	28	15.00	10.00	0.21	0.40
2	Annada	0.95	0.95	0.18	9.63	54.33	0.82	0.32	1.91	0.46	1.97	40	13.33	8.33	0.18	0.38
3	ARC 10372	0.04	0.85	0.95	22.00	84.67	0.72	0.26	5.18	1.15	2.87	60	11.00	20.00	0.26	0.65
4	AS 100	0.95	0.95	0.04	12.00	46.00	0.34	0.08	0.70	0.14	0.60	48	25.00	8.33	0.26	0.26
5	AS 1196	0.95	0.95	0.48	11.00	39.67	0.21	0.04	0.47	0.10	0.43	56	22.67	17.00	0.28	0.43
6	AS 178/3	0.95	0.95	0.18	16.17	61.33	0.41	0.08	2.57	0.63	1.03	40	14.33	4.67	0.27	0.25
7	AS 191	0.95	0.95	0.04	11.47	60.33	0.96	0.28	2.04	0.48	1.50	40	21.00	13.33	0.19	0.39
8	AS 193/1	0.95	0.95	0.85	11.03	44.33	0.47	0.31	0.94	0.32	0.80	60	9.00	40.00	0.25	0.81
9	AS 195	0.48	0.95	0.70	14.50	46.67	0.82	0.22	1.80	0.21	1.00	60	32.67	17.67	0.31	0.35
10	AS 203/2	0.75	0.85	0.18	14.00	62.33	0.78	0.15	0.82	0.26	1.20	60	9.33	7.00	0.23	0.44
11	AS 206	0.95	0.95	0.18	10.53	50.17	0.50	0.30	0.87	0.28	0.75	56	8.33	6.67	0.21	0.45
12	AS 208	0.95	0.95	0.95	13.50	42.33	0.39	0.10	0.77	0.16	0.55	52	12.67	6.33	0.32	0.33
13	AS 209	0.95	0.95	0.04	9.00	57.00	0.21	0.12	2.17	0.47	0.80	36	14.00	11.00	0.16	0.44
14	AS 229	0.95	0.95	0.70	11.23	61.33	0.36	0.24	1.78	0.52	0.60	48	20.33	10.00	0.18	0.33
15	AS 253	0.85	0.85	0.18	13.33	44.57	0.17	0.12	0.57	0.21	0.90	44	23.00	10.33	0.30	0.31
16	AS 27	0.95	0.95	0.70	11.00	61.33	0.74	0.12	0.86	0.22	1.07	52	22.33	12.33	0.18	0.36
17	AS 305	0.95	0.95	0.48	10.90	49.00	0.52	0.12	1.93	0.37	0.40	24	14.67	12.67	0.22	0.46
18	AS 313	0.48	0.95	0.04	17.00	77.67	1.64	0.35	7.47	1.49	2.13	24	16.67	6.33	0.22	0.27
19	AS 313/4	0.92	0.95	0.95	14.50	44.00	0.24	0.07	0.51	0.16	0.72	60	13.67	10.33	0.33	0.43
20	AS 314	0.55	0.55	0.48	9.90	68.33	0.25	0.12	2.21	0.53	0.75	40	15.67	13.00	0.15	0.45
21	AS 327	0.85	0.85	0.04	8.17	47.67	0.13	0.08	0.29	0.14	0.23	56	21.33	13.67	0.17	0.39
22	AS 36/20	0.95	0.95	0.95	10.93	53.00	0.94	0.37	1.96	0.45	1.78	40	11.33	17.00	0.21	0.60
23	AS 38/2	0.04	0.04	0.04	10.67	32.00	0.28	0.07	0.51	0.17	0.43	56	26.67	15.67	0.33	0.37
24	AS 39	0.95	0.95	0.95	15.00	51.67	0.41	0.12	0.78	0.26	0.50	52	30.00	15.00	0.29	0.33
25	AS 39/13	0.70	0.95	0.70	11.00	46.17	0.56	0.17	1.21	0.35	0.77	36	33.00	18.00	0.24	0.36
26	AS 48	0.80	0.95	0.04	11.83	51.00	0.16	0.06	0.94	0.21	0.53	60	17.33	8.00	0.23	0.31
27	AS 489	0.95	0.95	0.04	10.17	50.00	1.01	0.35	1.95	0.43	1.87	40	13.67	12.33	0.20	0.47
28	AS 64–65	0.95	0.95	0.85	17.00	55.00	0.38	0.12	0.74	0.26	0.55	44	31.00	11.00	0.31	0.26
29	AS 66–69	0.92	0.95	0.04	15.33	61.67	0.95	0.24	1.85	0.30	2.00	36	15.00	4.33	0.25	0.21
30	As 69–70	0.80	0.95	0.04	11.00	34.00	0.13	0.02	0.72	0.15	0.20	60	20.00	7.00	0.33	0.26
31	AS 75	0.95	0.95	0.48	13.00	73.67	1.70	0.29	3.80	0.64	2.50	52	11.00	5.33	0.18	0.32
32	AS 93/1	0.55	0.55	0.04	11.00	54.00	0.56	0.11	1.23	0.30	1.00	56	22.67	13.33	0.20	0.38
33	Bahmori	0.95	0.95	0.04	9.97	42.00	0.87	0.17	1.93	0.37	1.17	48	25.17	7.67	0.24	0.23
34	BairingGusmu	0.95	0.95	0.95	9.83	46.33	0.27	0.16	0.66	0.27	0.63	36	12.00	6.00	0.21	0.32
35	Balam -2	0.85	0.95	0.04	11.50	62.00	0.37	0.24	1.24	0.52	0.60	48	24.67	10.00	0.19	0.28
36	Banglami	0.04	0.92	0.48	14.33	75.33	0.95	0.18	2.61	0.57	2.07	56	28.00	16.00	0.18	0.36
37	Bangloni	0.75	0.92	0.48	10.17	56.33	0.75	0.13	1.85	0.36	1.63	28	21.33	11.00	0.18	0.34
38	BengenaGutia	0.95	0.95	0.95	11.00	57.67	0.44	0.13	1.21	0.34	1.07	56	38.00	15.33	0.19	0.29
39	Beria Bhonga	0.95	0.95	0.04	9.33	51.00	0.16	0.08	0.90	0.22	0.23	24	30.00	14.00	0.19	0.32
40	Bet GutiAhu	0.70	0.95	0.04	17.50	49.33	0.48	0.74	1.36	0.31	0.57	40	32.00	12.67	0.35	0.29
41	BilshaAhu	0.75	0.95	0.48	13.33	85.67	1.76	0.41	6.73	1.24	2.80	52	17.00	15.00	0.16	0.47
42	Bizor	0.04	0.04	0.04	14.87	50.33	0.42	0.16	3.68	0.73	1.43	60	21.00	6.00	0.30	0.24
43	Bizor-II	0.04	0.04	0.04	11.50	59.67	0.87	0.17	3.75	0.74	1.53	60	13.33	8.00	0.20	0.37
44	BogaAhu	0.85	0.95	0.04	10.50	54.33	0.47	0.06	1.08	0.19	1.00	56	16.00	10.00	0.19	0.38
45	Bogagajep	0.70	0.95	0.85	14.33	57.33	0.63	0.18	1.93	0.42	1.40	28	18.00	0.00	0.25	0.00
46	BongalAhu	0.95	0.95	0.95	13.60	51.33	0.55	0.13	1.20	0.18	1.40	52	20.33	10.00	0.27	0.32
47	Bor Kola Ahu	0.95	0.95	0.04	13.63	71.33	1.42	0.41	2.87	1.22	1.93	56	19.67	14.33	0.19	0.42
48	Chapali (144)	0.95	0.95	0.95	12.63	53.33	0.17	0.11	0.63	0.26	1.13	44	14.67	8.00	0.24	0.36
49	Chapali (264)	0.95	0.95	0.04	14.00	73.33	1.17	0.27	3.60	0.77	1.67	40	33.00	10.33	0.19	0.24
50	China	0.95	0.95	0.85	11.70	54.33	0.86	0.14	3.40	0.78	1.37	40	17.00	9.33	0.22	0.36
51	Darakhoj	0.70	0.95	0.04	9.50	61.33	0.81	0.25	2.73	0.75	1.00	52	16.00	4.33	0.16	0.21
52	Dau Kola Maghi	0.95	0.95	0.04	14.17	65.33	1.53	0.33	4.57	1.37	2.43	56	22.33	10.00	0.22	0.31
53	Decembor Sali-1	0.70	0.95	0.48	17.50	73.00	0.75	0.20	3.62	0.70	1.92	28	14.33	6.00	0.24	0.29
54	DogaRonga	0.95	0.95	0.04	15.33	50.00	0.91	0.21	1.87	0.30	2.00	40	15.00	6.00	0.31	0.28
55	DusriAhu	0.95	0.95	0.70	16.00	33.00	0.23	0.10	0.24	0.15	0.53	60	21.67	12.67	0.49	0.37
56	Gajef Sali-3	0.70	0.95	0.04	9.97	58.33	0.42	0.11	1.18	0.30	1.00	60	36.00	15.00	0.17	0.30
57	GaramAhu-II	0.95	0.95	0.04	10.00	39.00	0.16	0.03	0.45	0.09	0.38	56	19.67	17.33	0.26	0.47
58	Garem-I	0.95	0.95	0.04	19.23	61.83	0.28	0.11	1.55	0.41	0.75	52	26.00	9.33	0.31	0.26
59	GorunatiaAhu	0.80	0.95	0.95	18.00	83.83	1.54	0.28	3.72	0.73	2.63	60	14.67	9.33	0.22	0.39
60	GuborGuni	0.95	0.95	0.95	11.90	73.33	0.74	0.28	2.42	0.63	1.50	36	20.67	7.00	0.16	0.26
61	HafaAhu	0.95	0.95	0.95	22.67	74.00	2.65	0.34	4.89	1.06	4.00	40	24.33	8.33	0.31	0.26
62	Hafa Ahu-2	0.95	0.95	0.95	15.27	62.00	1.81	0.75	3.29	0.98	1.84	36	24.00	7.67	0.25	0.24
63	HaldharSali	0.85	0.95	0.04	16.83	61.00	0.75	0.20	1.00	0.38	1.43	56	28.33	6.33	0.28	0.18
64	HalodhiaGutiaSali	0.85	0.95	0.04	10.40	47.33	0.17	0.07	0.41	0.18	0.18	36	14.33	5.33	0.22	0.27
65	HatiSali	0.48	0.95	0.70	17.00	57.67	1.74	0.37	6.35	1.53	2.80	25	16.67	6.33	0.17	0.29
66	HaraipowaAhu	0.95	0.95	0.95	14.57	31.67	0.26	0.12	0.24	0.13	0.47	44	24.00	14.33	0.46	0.38
67	HorinKajuli	0.04	0.04	0.04	21.17	94.33	2.19	0.67	10.03	1.90	3.37	60	26.33	13.33	0.22	0.34
68	Hybrid-14	0.95	0.95	0.95	10.33	58.67	0.39	0.18	1.49	0.36	1.30	60	13.00	8.00	0.18	0.38
69	IkoraGuni	0.95	0.95	0.04	17.00	78.67	0.92	0.90	3.14	0.16	2.20	52	28.67	4.00	0.22	0.12
70	Ikora Guni-2	0.95	0.95	0.95	12.20	72.33	0.85	0.63	2.22	0.14	1.60	44	22.00	4.67	0.17	0.17
71	Inglongkiri	0.04	0.85	0.48	25.50	81.50	8.57	2.49	9.81	2.82	8.65	60	31.00	20.00	0.31	0.38
72	IR-64	0.95	0.95	0.85	10.50	44.33	0.92	0.20	1.99	0.42	1.20	36	30.00	12.00	0.24	0.29
73	Jahinga	0.48	0.95	0.04	11.20	60.67	0.29	0.16	1.59	0.54	0.78	24	34.33	13.67	0.18	0.29
74	KacherSali	0.95	0.95	0.95	11.13	79.33	0.70	0.28	2.41	0.63	1.74	56	41.00	15.00	0.14	0.26
75	Kehong	0.95	0.95	0.04	13.37	45.00	0.48	0.32	1.04	0.32	0.80	56	12.33	12.33	0.30	0.51
76	Kehong-2	0.95	0.95	0.85	18.07	62.67	0.75	0.14	1.91	0.45	0.77	40	18.33	4.67	0.29	0.20
77	Kholihoi	0.48	0.70	0.04	14.13	75.00	1.19	0.50	4.82	0.93	1.70	60	23.33	14.00	0.19	0.38
78	Koimurali	0.95	0.95	0.48	11.30	30.33	0.25	0.11	0.22	0.13	0.49	60	21.00	13.00	0.45	0.38
79	Koizapori-I	0.95	0.95	0.04	12.53	54.67	0.52	0.28	1.42	0.44	1.21	56	14.00	9.67	0.23	0.41
80	Koizapori-II	0.95	0.95	0.85	9.03	48.17	0.48	0.28	0.95	0.34	0.80	52	7.00	6.67	0.21	0.48
81	Koizapori-III	0.95	0.95	0.04	11.90	42.67	0.50	0.15	0.68	0.23	0.80	56	25.67	12.00	0.28	0.32
82	Kola Ahu	0.95	0.95	0.04	12.20	51.00	0.28	0.11	0.66	0.17	0.61	56	27.00	12.00	0.24	0.31
83	Kola Ahu-2	0.95	0.95	0.04	9.97	32.00	0.16	0.04	0.23	0.05	0.42	56	26.33	10.00	0.31	0.28
84	Kola MeghiAhu	0.95	0.95	0.48	10.90	66.67	0.24	0.10	1.18	0.27	0.83	32	22.67	10.00	0.16	0.30
85	Kola Meghi Ahu-2	0.95	0.95	0.95	9.00	60.00	0.18	0.05	1.40	0.28	0.80	28	25.00	12.00	0.15	0.32
86	Kola Sali	0.95	0.95	0.95	9.67	50.50	0.21	0.10	0.69	0.26	0.67	56	25.00	14.00	0.19	0.36
87	KoniAhu	0.92	0.95	0.95	13.17	53.00	0.90	0.15	1.30	0.27	1.53	36	39.00	25.00	0.25	0.40
88	Kosamoni	0.04	0.95	0.04	13.17	65.33	0.95	0.22	1.97	0.43	1.70	64	21.33	13.00	0.20	0.38
89	Lachit	0.55	0.70	0.70	14.50	30.00	0.39	0.19	0.52	0.18	0.41	40	18.00	4.00	0.23	0.18
90	LaudubiSali	0.95	0.95	0.95	8.00	49.50	0.11	0.08	0.41	0.24	0.50	60	41.33	10.00	0.16	0.19
91	Maijao	0.95	0.95	0.95	10.33	55.50	1.00	0.22	2.02	0.36	1.93	40	29.67	16.00	0.18	0.35
92	Maizao	0.95	0.95	0.04	12.50	40.00	0.80	0.20	1.75	0.41	0.70	36	32.00	16.00	0.31	0.33
93	ManoharSali	0.70	0.95	0.04	16.00	52.33	0.51	0.27	1.32	0.42	1.00	60	15.00	9.67	0.31	0.39
94	Mazu Bairon-2	0.70	0.70	0.04	14.50	60.67	0.90	0.13	2.00	0.40	1.20	48	33.67	7.33	0.24	0.18
95	MirenKillak	0.95	0.95	0.04	13.10	35.67	0.09	0.03	0.15	0.03	0.15	56	19.33	14.67	0.37	0.43
96	Naga Ahu	0.95	0.95	0.95	15.87	67.67	0.95	0.17	3.00	0.64	2.00	44	19.33	10.67	0.23	0.36
97	Nepali Sali	0.85	0.95	0.95	9.33	58.00	0.45	0.11	1.86	0.35	0.93	52	55.00	12.33	0.16	0.18
98	Nilazi	0.95	0.95	0.48	9.40	56.00	0.57	0.25	1.29	0.17	1.39	44	36.00	12.33	0.22	0.26
99	Norin 18/Patnai 23	0.70	0.95	0.04	10.67	60.33	0.55	0.22	1.42	0.43	1.00	16	6.67	9.33	0.18	0.59
100	PaniSali	0.70	0.95	0.48	8.33	52.00	0.27	0.05	1.99	0.45	0.90	52	8.67	11.00	0.16	0.56
101	PoromaAhu	0.95	0.95	0.95	11.03	61.13	0.51	0.22	1.42	0.36	0.57	56	33.67	7.00	0.21	0.17
102	Raja Ahu	0.04	0.95	0.04	10.00	30.00	0.30	0.10	0.80	0.09	0.53	56	16.00	10.67	0.34	0.40
103	Ranga Sali-2	0.95	0.95	0.95	7.00	51.00	0.36	0.10	0.40	0.19	0.33	16	23.00	11.00	0.16	0.32
104	Ranjit	0.95	0.95	0.04	12.17	45.00	0.62	0.13	1.68	0.36	1.30	24	31.00	11.67	0.27	0.29
105	Rikhoijoi	0.92	0.95	0.18	11.00	65.00	1.80	0.18	2.47	1.09	1.27	40	23.67	8.00	0.17	0.25
106	Rikhoijoi-2	0.95	0.95	0.69	10.27	58.67	0.69	0.18	0.62	0.31	1.43	48	20.50	10.17	0.17	0.33
107	RongaAhu	0.95	0.95	0.18	10.43	55.17	0.63	0.15	1.04	0.32	1.27	44	28.33	8.33	0.26	0.23
108	Ronga Doria-3	0.95	0.95	0.95	9.83	51.00	0.21	0.11	0.73	0.28	0.83	48	20.00	16.33	0.20	0.45
109	RongaJira	0.70	0.70	0.04	11.93	58.67	0.53	0.26	1.31	0.42	1.40	56	17.00	9.33	0.20	0.36
110	Rongadoria Local	0.55	0.75	0.48	13.07	67.00	0.49	0.27	1.23	0.36	0.79	52	25.00	17.67	0.20	0.41
111	RongKhong	0.95	0.95	0.48	12.73	65.50	0.58	0.25	1.48	0.38	0.63	56	20.33	13.00	0.19	0.39
112	Sercher	0.95	0.95	0.95	9.73	69.67	0.23	0.13	2.17	0.56	0.83	36	14.33	14.67	0.14	0.51
113	SohaliaAhu	0.95	0.95	0.95	12.00	74.33	0.75	0.28	2.35	0.65	1.47	40	17.00	7.00	0.16	0.29
114	SoruRongaAhu	0.95	0.95	0.04	10.63	33.33	0.16	0.06	0.26	0.08	0.53	60	29.33	9.33	0.32	0.25
	**Mean**	**0.82**	**0.89**	**0.43**	**12.60**	**56.03**	**0.70**	**0.22**	**1.86**	**0.45**	**1.20**	**49.66**	**21.96**	**11.10**	**0.23**	**0.34**
	**C.V**.	**4.13**	**2.29**	**9.68**	**7.37**	**6.21**	**41.16**	**69.63**	**33.11**	**46.54**	**51.02**	**14.90**	**20.85**	**26.20**	**8.34**	**20.53**
	**F ratio**	**137.36**	**243.15**	**262.18**	**36.10**	**44.14**	**27.38**	**8.65**	**21.63**	**10.38**	**7.97**	**12.40**	**10.03**	**8.53**	**35.22**	**7.41**
	**F Prob**.	**0.00**	**0.00**	**0.00**	**0.00**	**0.00**	**0.00**	**0.00**	**0.00**	**0.00**	**0.00**	**0.00**	**0.00**	**0.00**	**0.00**	**0.00**
	**S.E**.	**0.02**	**0.01**	**0.02**	**0.54**	**2.01**	**0.17**	**0.09**	**0.36**	**0.12**	**0.35**	**4.27**	**2.64**	**1.68**	**0.01**	**0.04**
	**C.D. 5%**	**0.05**	**0.03**	**0.07**	**1.49**	**5.60**	**0.47**	**0.25**	**0.99**	**0.34**	**0.99**	**11.90**	**7.36**	**4.68**	**0.03**	**0.11**
	**C.D. 1%**	**0.07**	**0.04**	**0.09**	**1.97**	**7.38**	**0.61**	**0.33**	**1.31**	**0.44**	**1.30**	**15.69**	**9.71**	**6.17**	**0.04**	**0.15**

### SSR genotyping

#### DNA isolation and PCR amplification

A total of 140 SSR markers and 30 genes specific (root related traits and aquaporin) SSR markers covering all 12 chromosomes were used to assess the level of genetic diversity among 114 rice accessions. Finally, 65 markers including four gene-specific markers out of 170 SSR markers were chosen for genetic diversity analysis because they were found polymorphic and showed prominent distinguishable banding patterns among genotypes. The genetic sequence of genes related to root traits and drought tolerance factors were downloaded from http://rapdb.dna.affrc.go.jp/ and http://rice.plantbiology.msu.edu/. Gene-specific primers were designed using Primer 3 software. SSR marker sequences, annealing temperature and chromosomal locations are obtained from the GRAMENE database. The total genomic DNA from each of the genotypes included in the present study was extracted following the protocol of Plaskhe *et al*.^46^ with slight modification. The quantity of genomic DNA was measured using a Nanodrop instrument. The final concentration of DNA was adjusted to 30 ng/μl for PCR reaction. The amplification conditions were based on the procedure of Panaud *et al*.^47^. The PCR reaction volume was 10 μl. The PCR reaction mixture of 10 μl consists of 0.4 mMdNTPs, 4 mM of MgCl2, 150 mM of Tris-HCl, 10 pmoles of forward and reverse primer and 0.05 U Taq polymerase with 30 ng of DNA. The reagents were mixed thoroughly and then placed in a Thermal Cycler (PCR Gene AMP® 2400, Applied Biosystems, USA) for cyclic amplification using the amplification programme Step 1 (Initial denaturation) 94 °C for 5 min. Step 2 (Denaturation) 94 °C for 1 min. Step 3 (Annealing) 32 °C for 1 min. Step 4 (Extension) 72 °C for 1 min. Step 5 (Final extension) 72 °C for 5 min. Step 6 (Storage) 4 °C for infinity. Steps 2, 3 and 4 were repeated 35 times.

#### Gel electrophoresis, photography and allele scoring

Amplified products were separated based on their size using 3% agarose gel electrophoresis and 1x TBE buffer in the horizontal electrophoresis tank. The photograph of the gel was digitally documented in Gel Documentation System (UVP, UK). The molecular weight of distinct bands or amplified fragments was measured in base pair by comparing with the band size of 100 bp ladder (GeNeI Company) with IR-36 as molecular weight reference^48^.

#### Data analysis

The root traits and drought tolerance were subjected to analysis of variance (ANOVA), clustering based on the algorithm of unweighted pair group method with arithmetic mean (UPGMA) and Principal Component Analysis (PCA) using R packages^49^.

To identify the genetic structure of the given population and assign individuals to populations, the software STRUCTURE version 2.3.4 was used^50^. To derive the optimal number of groups (K), STRUCTURE was run with K varying from 1 to 10, with five runs for each K value. To determine the true value of K, ad hoc statistic ΔK was followed. Parameters were set to 1,00,000 burn-in periods and 5,00,000 Markov Chain Monte Carlo (MCMC) replications after burn-in with an admixture and allele frequencies correlated model. The method described by Evano *et al*.^51^ was used to estimate the most probable K value for the analyzed data, using the web tool Structure Harvester ver. 0.6. application^52^.

The number of alleles (N), Ne (Allelic richness) Shannon information index (I), observed heterozygosity (Ho), expected heterozygosity (He), and fixation index (I)) were determined by using GenAlEx 6.502 programme^53^. A PIC value of each marker was determined as suggested by Botstein *et al*.^54^. Further, the allelic data were subjected to estimation of genetic distances among genotypes using simple matching coefficients by bootstrapping 1000 times and they were clustered using a neighbor-joining method using Darwin software version 6.0^55^. Further, analysis of molecular variance (AMOVA) was performed to describe variance components among individuals and the population differentiation among the seven assumed subpopulations using GeneAlEx 6.502 program^53^ with 1000 permutations. Principal coordinate analysis (PCoA) was performed to highlight the resolving power of the ordination and the first two components were used to represent the genotypes in the graphical form. PCoA and dissimilarity matrix was performed by using DARwin software version 6.0^55^. Genetic differentiation among the assumed subpopulation was analysed using Nei’s gene diversity statistics using GenAlEx program version 6.502. Venn diagram analysis was performed to identify common varieties between the model-based cluster and neighbor-joining based grouping using online interactive tool Venny 2.1^56^. A Mantel test was done to test similarities of distance and kinship matrices, with 30,000 permutations for a two-tailed Mantel test using R packages^57^.

## Result

### Phenotypic traits

**Figure 1:**
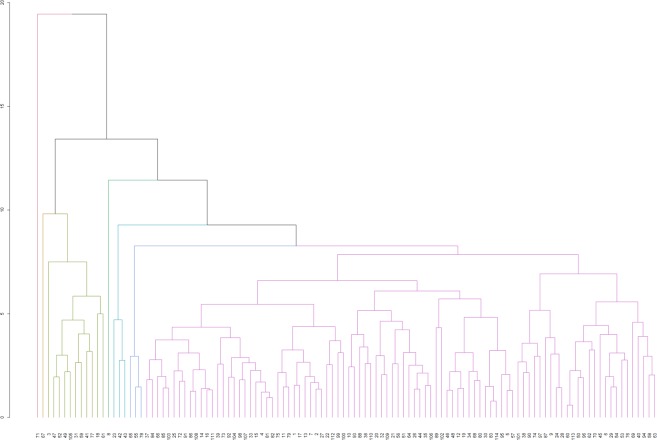
Grouping of genotypes based on the root, shoot and drought tolerance traits using UPGMA. Note: Numbering in x-axis indicates the genotypes serial number in Table 1.

Analysis of variance revealed significant differences among the genotypes for all traits measured in the study (Table 2). UPGMA based dendrogram analysis grouped the 114 rice accession into 7 clusters using fifteen roots and shoot traits. Clusters III, V, VI and VII comprised of 11, 3, 3 and 97 rice germplasm, respectively (Fig. 1). The cluster I composed a solitary genotype ‘Inglongkiri’ which was the best genotype among 114 rice germplasm considering several root traits together. The cluster II composed of ‘Horin Kajuli’ which showed drought tolerance with high shoot and fresh shoot weight. The cluster III genotypes have a relatively low root angle. The genotypes ‘ARC10372’ and ‘As 313’ were the best genotypes in this cluster for root and shoot traits. The cluster IV was composed of ‘As 1913/1’ which was found to be drought susceptible with the shallow and fibrous root system. The cluster V composed of ‘As 138/2’, ‘Bizor’ and ‘Bizor-II’ genotypes which showed to be drought tolerant. Genotypes of cluster VI were dwarf genotypes with the shallow but narrow root system. The cluster VII was the largest group with 97 genotypes, among them ‘As 314’, ‘As 93/1’, ‘Jahinga’, ‘Kosamoni’ and ‘Raja Ahu’ showed drought tolerance. These results revealed that considerable variation existed among genotypes for drought, root and shoot traits.

**Table 2:** ANOVA of 114 genotypes for root, shoot and drought tolerance parameters.

Source of Variation	df	Mean square														
		Drought Score at 32 DAWW	Drought Score at 34 DAWW	Recovery	Root Length	Shoot Length	Fresh Root weight	Dry Root weight	Fresh Shoot Weight	Dry Shoot Weight	Root Volume	Root Angle	Peripheri Root	Bottom root No.	Root:Shoot ratio	Ratio of Deep Rooting
Replicate	2	0.000	0.000	0.002	5.429	31.840	0.106	0.035	0.311	0.079	0.412	6.161	28.167	6.527	0.000	0.010
Treatments	113	0.159^*^	0.102^*^	0.464*	31.094*	534.077*	2.302*	0.206*	8.245*	0.451*	3.001*	678.903*	210.243*	72.137*	0.013*	0.036*
Error	226	0.001	0.000	0.002	0.861	12.100	0.084	0.024	0.381	0.043	0.377	54.736	20.951	8.453	0.000	0.005

^*^Significant at 1%.

**Figure 2:**
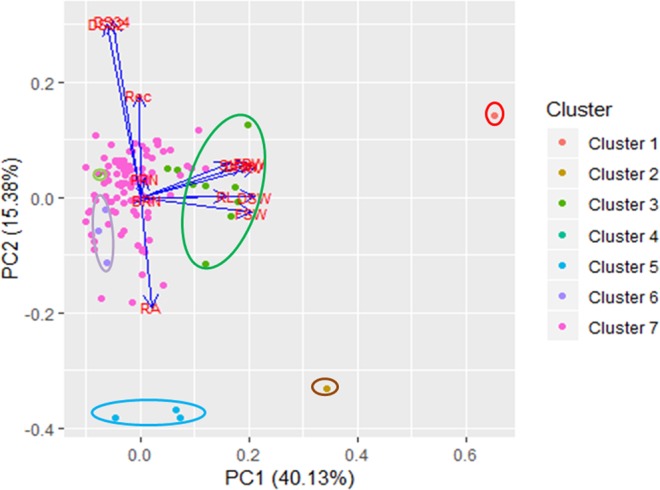
Grouping of genotype based on the first two principal components.

PCA analysis revealed that the first principal component with eigenvalue of 5.21 accounted for 40.13% of contribution to total variation and a second component with eigenvalue of 2.0 accounted for 15.38% of contribution to the total variation in the population. These two principal components include fresh shoot weight, root volume, dry shoot weight, fresh root weight, drought score after 32 and 34 DAWW (Fig. 2).

### SSR polymorphism among rice varieties

The polymorphism information content (PIC) of markers along with allele information such as number, size, highest frequency detected among 114 accessions is presented in Table 3. In the present diversity analysis, PIC value ranged from 0.005 for RM 87 to 0.802 for RM 418 with an average of 0.51 for all the genotypes under study (Table 3). The primer RM 474 and RM 320 showed higher discriminatory power to distinguish genotypes due to its high PIC value 0.747 and 0.749 respectively. The primer RM 87 and RM 480 showed lower PIC value 0.005 suggesting less discriminatory power of this primer under study. PIC value of microsatellite marker higher than 0.5 is considered highly informative^54^. The highest resolving power (3.05) was observed for RM219 and lowest (0.32) for RM480 with an average of 1.37. The most major frequent allele frequency of 0.90 was observed for the marker RM480 and the lowest (0.35) was observed for RM24 with a mean of 0.61.

**Table 3:** Polymorphism information observed among 114genotypes based on SSR markers.

Sl. No.	Primer	Chromosome No.	Annealing temp	Allele No.	Size range (bp)	Highest frequency		PIC
						Size (bp)	Freq (%)	
1	OsSIP1;1	2	53	2	110–115	115	0.45	0.66
2	P5CS (Proline)	1	55	2	230–240	230	0.84	0.28
3	OsZIP33	3	55	2	220–240	240	0.70	0.44
4	SnRK2	3	53	2	220–240	240	0.68	0.47
5	RM423	2	55	2	270–295	270	0.67	0.49
6	RM424	2	55	3	240–295	260	0.45	0.66
7	RM475	2	55	2	185–200	200	0.53	0.57
8	RM422	3	55	2	370–385	385	0.50	0.57
9	RM127	4	55	2	210–230	230	0.61	0.55
10	RM567	4	55	2	240–260	240	0.73	0.44
11	RM480	5	55	2	190–225	225	0.90	0.18
12	RM592	5	55	4	200–400	270	0.37	0.72
13	RM400	6	55	3	195–280	220	0.43	0.64
14	RM336	7	55	3	125–195	148	0.42	0.67
15	RM418	7	55	2	250–300	300	0.41	0.80
16	RM210	8	55	2	140–165	140	0.49	0.57
17	RM474	10	55	2	226–256	256	0.36	0.75
18	RM566	9	55	2	230–300	300	0.44	0.72
19	RM333	10	55	3	165–215	192	0.56	0.63
20	RM496	10	55	2	290–265	291	0.62	0.52
21	RM21	11	55	2	132–170	132	0.68	0.49
22	RM167	11	55	2	127–159	127	0.65	0.49
23	RM206	11	55	3	128–202	128	0.50	0.66
24	RM9	1	55	3	124–194	194	0.75	0.41
25	RM24	1	55	3	152–198	198	0.35	0.70
26	RM551	4	55	3	170–240	192	0.47	0.66
27	RM3	6	55	3	110–150	120	0.69	0.51
28	RM276	6	55	2	85–150	85	0.75	0.40
29	RM228	10	55	2	108–154	108	0.57	0.67
30	RM429	7	55	2	140–185	140	0.72	0.43
31	RM6	2	55	2	150–165	165	0.68	0.51
32	RM48	2	55	2	200–220	200	0.82	0.24
33	RM22	3	55	2	185–200	200	0.54	0.54
34	RM128	1	55	2	150–165	150	0.66	0.48
35	RM8	2	55	2	250–160	250	0.62	0.57
36	RM13	5	55	2	130–150	130	0.71	0.45
37	RM87	5	55	2	125–130	150	0.82	0.01
38	RM161	5	61	2	165–190	165	0.65	0.54
39	RM249	5	55	2	120–150	150	0.61	0.48
40	RM1	1	55	3	65–120	70	0.58	0.62
41	RM495	1	55	2	130–140	140	0.88	0.22
42	RM431	1	55	2	250–270	270	0.54	0.53
43	RM212	1	55	2	125–145	125	0.81	0.32
44	RM263	2	55	2	170–195	195	0.53	0.51
45	RM7332	3	55	2	130–150	150	0.69	0.50
46	RM22	3	55	2	195–200	200	0.52	0.52
47	RM545	3	55	2	210–230	210	0.68	0.44
48	RM252	4	55	3	150–170	150	0.62	0.53
49	RM127	4	55	2	122–130	130	0.55	0.50
50	RM241	4	55	2	100–110	100	0.54	0.65
51	RM440	5	55	2	170–220	170	0.61	0.48
52	RM320	7	55	3	210–230	220	0.40	0.75
53	RM125	7	55	2	125–140	125	0.68	0.45
54	RM25	8	55	2	130–140	130	0.54	0.60
55	RM72	8	55	2	160–180	160	0.41	0.59
56	RM152	8	55	2	125–140	125	0.66	0.46
57	RM408	8	55	2	120–140	120	0.81	0.32
58	RM219	9	55	4	130–230	200	0.60	0.21
59	RM24334	9	55	2	120–130	130	0.75	0.38
60	RM24390	9	55	2	80–90	80	0.67	0.47
61	RM171	10	55	2	320–370	320	0.69	0.43
62	RM202	11	55	3	180–190	180	0.60	0.56
63	RM19	12	55	2	200–230	200	0.66	0.46
64	RM28166	12	55	2	200–210	200	0.60	0.50
65	RM12	12	55	2	170–200	170	0.75	0.38

Note: Major allele is described as the allele with the highest frequency.

### Population genetic diversity

The population-level genetic diversity of the rice accessions under study is presented in Table 4. Altogether 147 alleles were detected using 65 SSR markers, with an average of 2.26 alleles per locus, which indicated that genotypes of the present study were diverse. The number of alleles amplified varied from 2–4 and the highest number of alleles (4 alleles) were detected for RM 219 and RM 592. These two markers RM 219 and RM 592 showed the highest Nei’s Gene diversity of 0.658 and 0.601 respectively. Observed heterozygosity (H_o_) ranged from 0 to 0.725 (RM219) with an average of 0.036 across all 65 loci. The majority of the SSR markers exhibited observed heterozygosity as zero, indicating that the majority of rice germplasm used in the present study were pure and completely homozygous for SSR markers used in the present study, which may be the result of the self-pollinated mode of reproduction of rice. Observed heterozygosity (0.041) was far lower than total expected heterozygosity (0.467) which is further supported by low gene flow (Nm) value for the majority of loci, except RM87, RM431, RM333, RM 336 and RM495. The average value of Nm was recorded at 3.264. Expected heterozygosity or gene diversity (He) estimated based on Nei distance varied from 0.118 (RM480) to 0658 (RM219) with an average of 0.334.

**Table 4:** Genetic diversity of 65 SSR markers in the 114 rice genotypes.

Marker	Na	Ne	Ht	He	Ho	F_IS_	F_IT_	F_ST_	Nm	PIC
OsSIP1;1	2	2.483	0.455	0.405	0	1	1	0.111	2.012	0.66
P5CS (Proline)	2	1.437	0.366	0.163	0	1	1	0.556	0.2	0.28
OsZIP33	2	1.490	0.473	0.190	0.02	0.897	0.959	0.598	0.168	0.44
SnRK2	2	1.915	0.468	0.311	0	1	1	0.335	0.496	0.47
RM423	2	1.627	0.498	0.245	0	1	1	0.508	0.242	0.49
RM424	3	2.525	0.656	0.537	0.008	0.985	0.988	0.182	1.125	0.66
RM475	2	1.892	0.456	0.319	0.008	0.976	0.983	0.299	0.586	0.57
RM422	2	2.023	0.439	0.331	0.009	0.973	0.98	0.245	0.769	0.57
RM127	2	1.818	0.439	0.283	0.009	0.968	0.98	0.355	0.454	0.55
RM567	2	1.958	0.411	0.353	0	1	1	0.14	1.538	0.44
RM480	2	1.313	0.132	0.118	0.023	0.809	0.829	0.107	2.079	0.18
RM592	4	3.631	0.673	0.601	0.122	0.797	0.819	0.107	2.083	0.72
RM400	3	3.503	0.663	0.618	0.399	0.354	0.398	0.068	3.442	0.64
RM336	3	2.988	0.577	0.503	0.044	0.912	0.924	0.128	1.7	0.67
RM418	2	2.242	0.401	0.373	0.01	0.973	0.975	0.069	3.37	0.8
RM210	2	2.091	0.502	0.346	0.005	0.985	0.99	0.311	0.553	0.57
RM474	2	2.325	0.492	0.363	0	1	1	0.261	0.708	0.75
RM566	2	2.743	0.479	0.478	0	1	1	0.002	104.6	0.72
RM333	3	2.627	0.49	0.469	0	1	1	0.043	5.616	0.63
RM496	2	1.739	0.47	0.287	0	1	1	0.389	0.393	0.52
RM21	2	1.958	0.477	0.288	0	1	1	0.396	0.381	0.49
RM167	2	1.688	0.34	0.293	0	1	1	0.14	1.54	0.49
RM206	3	2.673	0.653	0.546	0	1	1	0.164	1.275	0.66
RM9	3	2.142	0.569	0.312	0.202	0.352	0.645	0.453	0.302	0.41
RM24	3	2.785	0.662	0.538	0.031	0.943	0.953	0.187	1.09	0.7
RM551	3	2.397	0.6	0.446	0.037	0.916	0.938	0.257	0.724	0.66
RM3	3	1.986	0.538	0.2	0	1	1	0.628	0.148	0.51
RM276	2	1.747	0.36	0.225	0.025	0.889	0.931	0.374	0.419	0.4
RM228	2	2.049	0.249	0.205	0	1	1	0.178	1.157	0.67
RM429	2	1.640	0.292	0.267	0.023	0.912	0.92	0.084	2.711	0.43
RM6	2	1.557	0.437	0.123	0	1	1	0.719	0.098	0.51
RM48	2	1.811	0.362	0.329	0.293	0.112	0.192	0.09	2.513	0.24
RM22	2	1.839	0.499	0.28	0	1	1	0.439	0.32	0.54
RM128	2	1.646	0.474	0.257	0	1	1	0.458	0.296	0.48
RM8	2	2.086	0.336	0.318	0	1	1	0.053	4.479	0.57
RM13	2	1.669	0.414	0.249	0	1	1	0.398	0.377	0.45
RM87	2	2.235	0.437	0.428	0.441	−0.03	−0.007	0.022	11.2	0.01
RM161	2	1.676	0.449	0.147	0	1	1	0.674	0.121	0.54
RM249	2	1.894	0.499	0.361	0.18	0.501	0.639	0.276	0.655	0.48
RM1	3	2.321	0.536	0.466	0	1	1	0.131	1.658	0.62
RM495	2	1.432	0.196	0.193	0	1	1	0.015	16.599	0.22
RM431	2	2.137	0.495	0.479	0	1	1	0.034	7.087	0.53
RM212	2	1.759	0.433	0.266	0	1	1	0.385	0.399	0.32
RM263	2	1.944	0.496	0.338	0.005	0.986	0.99	0.318	0.536	0.51
RM7332	2	1.807	0.191	0.166	0.006	0.967	0.971	0.129	1.69	0.5
RM22	2	1.881	0.496	0.327	0	1	1	0.34	0.486	0.52
RM545	2	1.767	0.393	0.369	0	1	1	0.062	3.766	0.44
RM252	3	2.502	0.594	0.521	0.005	0.991	0.992	0.123	1.784	0.53
RM127A	2	2.011	0.507	0.434	0.016	0.964	0.969	0.144	1.49	0.5
RM241	2	1.914	0.719	0.231	0	1	1	0.678	0.119	0.65
RM440	2	1.442	0.488	0.19	0	1	1	0.611	0.159	0.48
RM320	3	2.980	0.589	0.472	0	1	1	0.199	1.004	0.75
RM125	2	1.608	0.312	0.232	0	1	1	0.257	0.724	0.45
RM25	2	1.917	0.491	0.267	0	1	1	0.455	0.3	0.6
RM72	2	2.365	0.495	0.441	0	1	1	0.109	2.035	0.59
RM152	2	1.764	0.484	0.378	0	1	1	0.219	0.892	0.46
RM408	2	1.464	0.445	0.144	0	1	1	0.678	0.119	0.32
RM219	4	3.275	0.719	0.658	0.725	−0.102	−0.008	0.085	2.7	0.21
RM24334	2	1.696	0.347	0.288	0	1	1	0.169	1.227	0.38
RM24390	2	1.768	0.438	0	0		1	1	0	0.47
RM171	2	1.727	0.456	0.287	0.005	0.983	0.989	0.37	0.425	0.43
RM202	3	2.600	0.631	0.524	0	1	1	0.17	1.224	0.56
RM19	2	1.800	0.455	0.262	0.026	0.9	0.942	0.425	0.338	0.46
RM28166	2	1.886	0.453	0.362	0	1	1	0.202	0.988	0.5
RM12	2	1.605	0.334	0.303	0	1	1	0.093	2.444	0.38
Mean	2.26	2.05	0.467	0.334	0.041	0.905	0.921	0.28	3.264	0.51

Na = Number of alleles, Ne = Number of effective alleles, Ht = Total expected hetrogygosity, He = Gene Diversity, Ho = Observed Hetrogygosity, FIS = inbreeding coefficient, FIT = inbreeding coefficient to total, FST = Fixation index and Nm = Gene flow.

### Genetic relationship among the germplasm

#### Population structure analysis using the model-based approach

**Figure 3:**
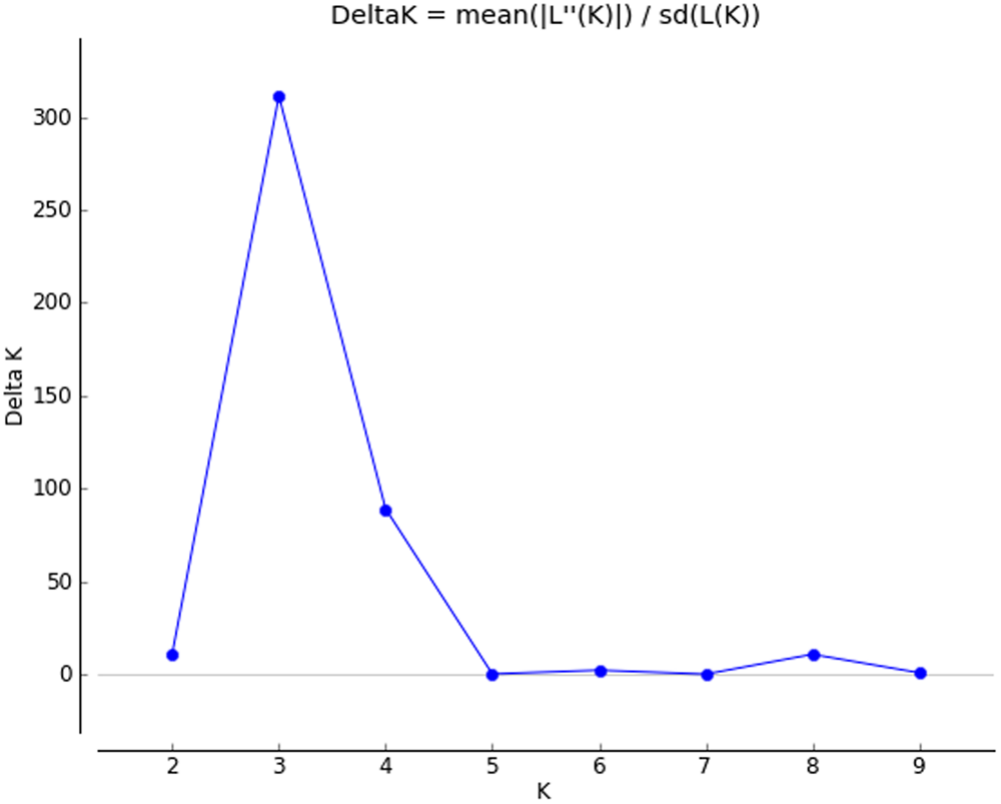
A plot of delta K values from the Structure analyses of 114 rice accessions, obtained through Structure harvester ver. 0.6. Application (Earl and Vonholdt^52^).

**Figure 4:**
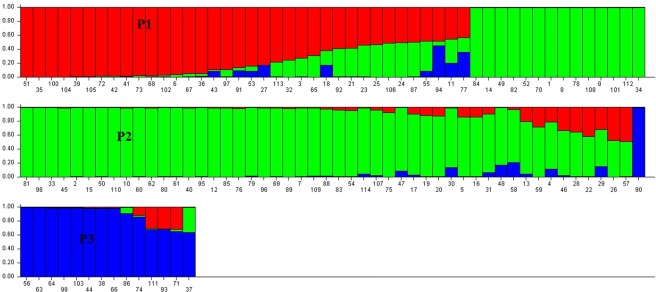
Population structure of 114 rice accession based on 65 SSR markers. Note: Numbering of genotypes corresponds to the serial number in Table 1.

In the present study grouping in population was determined using STRUCTURE analysis. The population structure of the 114 genotypes was analyzed by Bayesian clustering model-based approach with admixture and k value ranging from 1 to 10 with 5 iterations using 65 polymorphic markers. The ΔK was found highest for the model parameter K = 3 then for other value of K (Fig. 3) and the standard deviation was least at K = 3. Hence the true number of subpopulations were considered as three (P1, P2 and P3) (Fig. 4) which indicated that the whole population can be stratified into three subpopulations. Based on the sharing of genomic regions, genotypes in different populations were classified as pure or admixture. The accessions with the probability of ≥80% were considered as pure and assigned to corresponding subgroups while <than 80% were categorized as admixture (Fig. 4). Among 114 genotypes, 84 were pure and 30 rice accessions were admixture. Subpopulation P1 showed 21 pure (58.3%) and 15 admixed (41.6%) landraces, P2 had 52 pure (82.5%) and 11 (17.5%) admixed landraces, and P3 had 11 pure (73.3%) and 4 (26.7%) admixed individuals. No significant grouping was observed based on drought tolerance and roots and shoot trait data in the present study. However, the P1 population has ‘Kosamoni’ as a tolerant genotype having the highest root angle (64°) indicating a narrow rooting system. The best performing genotypes in the population (P1) were ‘Kosamoni’ for drought tolerance and ‘Hafa Ahu’ for root volume, fresh root weight and root length. The genotypes ‘Banglami’, ‘Bizor’, ‘Bizor-2’, ‘Horin Kajuli’ and ‘Raja Ahu’ genotypes were the best genotypes of the P2 population for drought tolerance and high recovery rate. ‘Horin Kajuli’ was the best genotype of this group in terms of drought tolerance, root volume, fresh root weight and dry shoot weight. In this group, ‘Boga Gajeb’, ‘Gajef Sali-3’ and ‘Norin 18/Patnai 23’ showed moderate drought tolerance and rest genotypes were susceptible to drought. As many as, 30 genotypes were observed as admixtures. Among the genotypes of admixtured origin, the genotypes ‘Inglongkiri’, ‘ARC 10372’, ‘As 38/2’ showed drought tolerance. Among all 114 genotypes, ‘Inglongkiri’ was also found to be the best genotypes for root volume, fresh root weight and dry shoot weight and root length. This indicated that different population identified using SSR markers also showed variation for different traits under study. These drought-tolerant genotypes can be used in drought tolerance improvement breeding programmes in rice.

F_ST_ statistics were calculated using STRUCTURE software to estimate the level of population structure. The F_ST_ values of 0.319, 0.332 and 0.570 for sub-populations P1, P2 and P3 with an average value of 0.407 indicated moderate population structure. In model-based analysis mean alpha value was 0.1022. The genetic differentiation among subpopulations was very high (average F_ST_ = 0.407) based on classification given by Wright^58^. Among the subpopulations, the P3 was highly differentiated followed by the P2 and P1. This genetic differentiation might be a result of natural selection favouring a different set of alleles in different ecologies and physical barrier which leads to low interchange or migration of alleles between different subpopulations. This is further supported by low gene flow (Nm) value for the majority of loci, except RM566 and RM495 SSR marker gene flow value. The average distance between individuals in P1, P2 and P3 was observed 0.358, 0.323 and 0.240 respectively, which indicated that P1 was the most diverse and less differentiated as compared to P2 and P3 subpopulation.

Pairwise F_ST_ values of sub-population range from 0.152 to 0.222 and showed significant differentiation among all the pairs which suggested that all the three groups were significantly different from each other. Based on pairwise F_ST_ estimate, P2 and P3 showed the highest level of differentiation from each other and population P1 and population P2 exhibited less differentiation from each other (Table 5).

**Table 5:** Pairwise population differentiation (F_ST_ value) below diagonal and gene flow (Nm) values above diagonal.

Population	P1	P2	P3
P1	0.000	1.398	1.097
P2	0.152	0.000	0.875
P3	0.186	0.222	0.000

The genetic diversity at the subpopulation level was studied in terms of the mean number of alleles (Na), No of effective alleles (Ne), observed heterozygosity (Ho), gene diversity (He), unbiased expected heterozygosity (uHe) and Wright’s fixation index (F), which is presented in Table 6.

**Table 6:** Genetic diversity statistics of 114 rice genotypes at sub-population levels

Pop	Na	Ne	I ± SE	Ho ± SE	He ± SE	uHe ± SE	F
P1	3.292	2.227	0.889 ± 0.029	0.036 ± 0.013	0.516 ± 0.016	0.523 ± 0.016	0.938 ± 0.021
P2	3.169	1.904	0.728 ± 0.037	0.029 ± 0.014	0.423 ± 0.022	0.426 ± 0.022	0.95 ± 0.022
P3	3.062	2.018	0.82 ± 0.029	0.043 ± 0.019	0.469 ± 0.017	0.486 ± 0.017	0.931 ± 0.031

No. of different alleles (Na), No. effective alleles or allelic richness (Ne), Shanon information Index (I), observed heterozygosity (Ho), gene diversity (He), unbiased expected heterozygosity (uHe) and F fixation index (F).

The Na, F and Ne were comparable among the three subpopulations (Table 6). The gene diversity was highest in P1. In all subpopulations, the mean expected heterozygosity was higher than mean observed heterozygosity. This was supported by the Mean fixation index of all subpopulations which was varied from 0.819 to 0.914.

#### Analysis of molecular variance (AMOVA)

AMOVA was done on population provided by model-based analysis, because of its reliability and consistency to provide detail information about the genetic constitution of the population. AMOVA revealed the presence of 17% of the variation was among populations, whereas, 77% of the variation among individuals and 6% of the variation among individuals within a population (Table 7). AMOVA revealed that most of the variation in rice accession mainly occurred among individuals. Wright’s F statistic (F_ST_) was 0.174, while F_IS_ and F_IT_ were 0.93 and 0.94, respectively. Higher F_IS_, which is measured at the subgroup level in the whole population, has indicated a lack of heterozygosity and high distinctness of populations, due to the autogamous nature of the crop. Determination of F_ST_ has shown high genetic variation among the population.

**Table 7:** Analysis of molecular variance (AMOVA) of 114 rice genotypes.

Source	Df	SS	MS	Est. Var.	% of variation
Among Pops	2	486.090	243.045	3.241	17%
Among Individual	111	3289.690	29.637	14.283	77%
Within Individual	114	122.000	1.070	1.070	6%
Total	227	3897.781		18.595	100%
**F-Statistics**	**Value**	**P(rand > = data)**			
F_ST_	0.174	0.001			
F_IS_	0.930	0.001			
F_IT_	0.942	0.001			
Nm	1.184				

Nei genetic distance ranged from 0.190 to 0.333. The maximum distance was observed between Pop 3 and pop 1 (0.333) and minimum distance was observed between pop1 and pop 2 (0.190), indicating that genomic differences between pop 3 and pop1 were more and it was less between pop1 and pop2 (Table 8).

**Table 8:** Pairwise population matrix of Nei genetic distanceof 114 rice genotypes at sub-population levels.

Populations	P1	P2	P3
P1	0.000		
P2	0.190	0.000	
P3	0.333	0.303	0.000

#### Neighbor-joining based clustering

**Figure 5:**
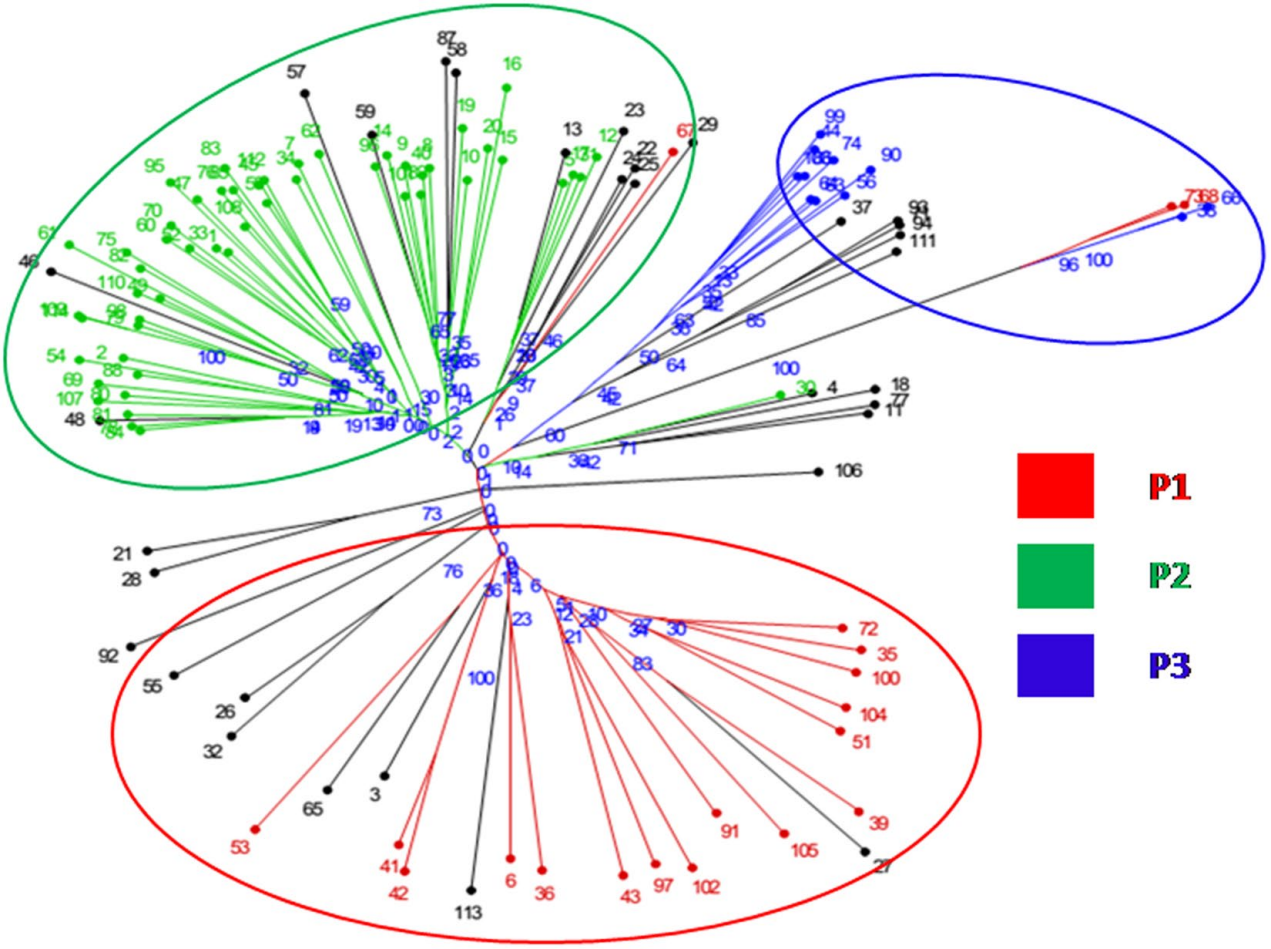
Unrooted neighbor-joining tree of 114 rice genotypes using SSR markers.

**Figure 6:**
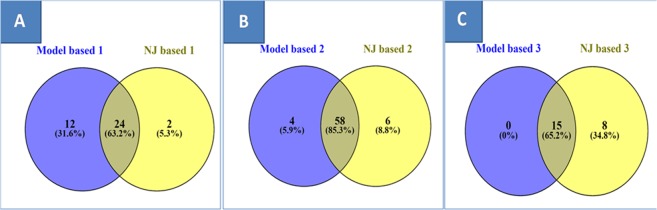
(**A**) Venn diagram showing co-linearity between model-based sub-population P1 & neighbor-joining based cluster 1. (**B**) Venn diagram showing co-linearity between model-based subpopulation P2 & neighbor-joining based cluster 2. (**C**) Venn diagram showing co-linearity between model-based subpopulation P3 & neighbor-joining based cluster 3.

An unweighted neighbor-joining tree, based on the alleles detected by 65 SSR markers, showed the genetic relationships among the 114 accessions. Cluster analysis based on the unweighted neighbor-joining clustering method separated the accessions into three main groups along with admixture genotypes spreading over three different clusters (Fig. 5), which showed similar results as model-based analysis. Cluster I was the largest cluster consist of 64 genotypes. Cluster II consisted of 26 genotypes followed by Cluster III having 24 genotypes. This pattern of clustering confirmed the existence of a significant amount of diversity. The genetic relationship results of the model-based analysis were compared with the unweighted neighbor-joining clustering method using the Venn diagram. Cluster-I generated through neighbor-joining unweighted analysis has 63.2% similarity of genotypes with subpopulation P1 generated through model-based analysis Fig. 6(A), whereas cluster-II showed 85.3% correspondence with subpopulation 2, Fig. 6(B). Similarly, cluster-III showed 65.2% of similarity of genotype sharing with subpopulation 3, Fig. 6(C).

#### Principal coordinate analysis

**Figure 7:**
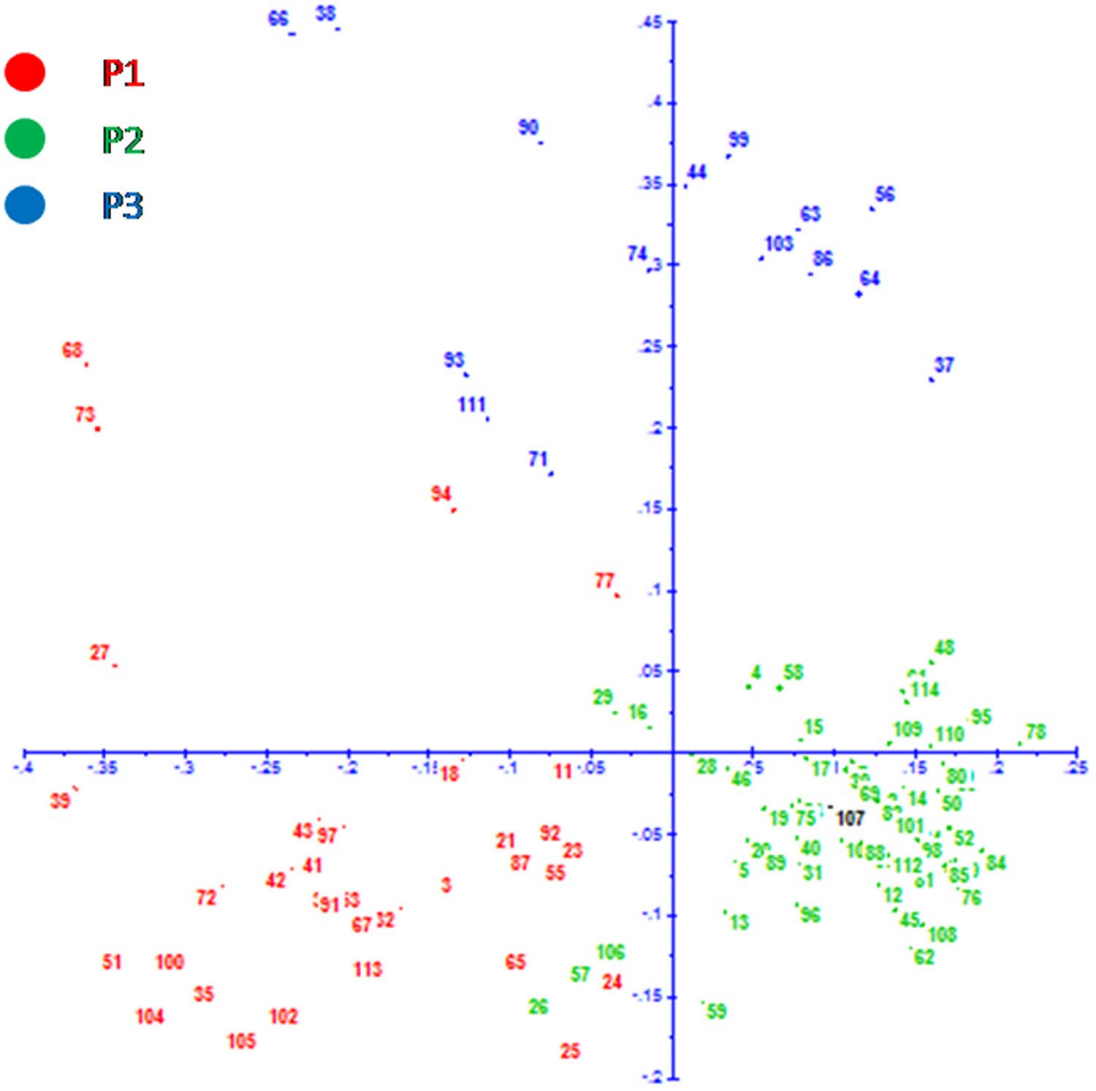
Principal Coordinate Analysis of 114 rice genotypes using SSR markers. Note: Numbering of genotypes corresponds to the serial number in Table 1.

PCoA using SSR markers allelic data determines the genetic relatedness among the genotypes. The first three axes of differentiation explained 27.87% of the total variation. The first coordinate explained 11.63% of the variation and second coordinate explained 9.12% of the variation (Fig. 7). PCoA analysis, similarly, grouped the genotypes as that of model-based analysis.

**Figure 8:**
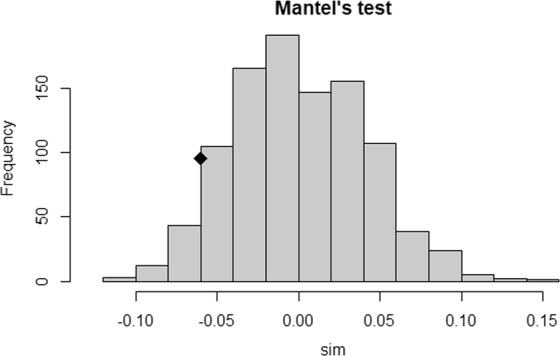
Mantel test plot showing genotypic vs phenotypic distance (P = 0.937).

The results of unweighted neighbor-joining clustering tree and PCoA analysis were in close correspondence to results of model-based STRUCTURE analysis which further confirmed the population STRUCTURE results. The histogram showing the sampling distribution of our 1000 randomly-produced Pearson correlations with the diamond symbol showed the location of our observed correlation (0.059) (Fig. 8). Mantel test P value (0.937) revealed a lack of correlation between genotypic and phenotypic distances.

A close correspondence was recorded in the results of AMOVA and F_ST_ analysis with that obtained from model based analysis, PCoA and unweighted neighbor-joining clustering. These revealed that the population under study has high genetic diversity and moderate population structure.

## Discussion

Analysis of genetic relationship among individual is an important component and play a major role in their effective utilization in the crop improvement programme. Genetic diversity study provides knowledge about the level of genetic diversity and genetic structure of the population and serve as a platform for the selection of superior genotypes to be used as parents in crop improvement breeding programmes. The popularization of few improved varieties in a crop like rice among the farming community leads to the narrow genetic base of crop species which results in high susceptibility of crops to various abiotic and biotic stress damages^59,60^. Therefore the inclusion of diverse valuable genetic base in breeding programmes can play a key role in the improvement of the degree of tolerance against various abiotic and biotic stress damages. The present investigation was aimed to throw some light on genetic variation for root traits and genetic diversity in North East Indian rice genotypes for their effective utilization in the breeding programme.

Dendrogram analysis based on root, shoot and drought tolerance traits revealed that the clustering pattern obtained is determined by mainly fresh shoot weight, root volume, dry shoot weight, fresh root weight and drought score traits. Cluster I genotype was characterized by the highest root volume, fresh root weight and dry root weight. Cluster II genotype is characterized by the highest fresh shoot weight and drought tolerance. Cluster IV genotype was characterized by drought susceptibility, root length and bottom root number. Cluster V is characterized by high drought tolerance and cluster 6 is characterized by low value for fresh shoot weight, dry shoot weight, root volume and fresh root weight. Cluster VII is characterized by drought susceptibility and intermediate value for root volume, fresh root weight, fresh shoot weight and dry shoot weight. The PCA analysis revealed that maximum diversity in a population of 114 rice germplasm was governed by fresh shoot weight, root volume, dry shoot weight, fresh root weight, drought score traits. This study revealed sufficient diversity and genotypes identified to be superior for one or more traits from different clusters might be useful in the hybridization programme to identify desirable segregants for the traits under study.

Genetic diversity refers to the presence of contrasting alleles of a gene in different individuals of the same species^61,62^. Diversity analysis using molecular markers is advantageous over the conventional approach based on phenotypic data, as molecular markers provide true information at a genetic level without the influence of environmental effects and provide information about the genetic constitution of genotypes such as which genomic regions or alleles have come from which population^63^. Genetic diversity study provides not only the phylogenetic relationship but also provide a chance of a finding a new and useful novel alleles present in a diverse set of accessions^64^.

Polymorphism information content (PIC) indicates the informativeness of a marker and allelic diversity of the population. PIC value of 1 indicates that marker is highly polymorphic, and would have an infinite number of alleles, and the marker is more informative, suggesting higher discriminatory power of marker^65^. SSR polymorphism analysis revealed an average PIC of 0.507 for 65 markers, which reflected the better discriminatory power of these markers to reveal the higher level of genetic diversity among genotypes and indicated the diverse nature of accessions under study. Similar results for average PIC were reported by Das *et al*.^66^ in the landraces of northeast India. Behera *et al*.^67^ reported a higher average PIC of 0.811 per locus, this might be due to the use of a more diverse set of rice accession in their study or due to the use of highly polymorphic markers. Shah *et al*.^68^ and Pachauri *et al*.^69^ have reported mean PIC values 0.37 and 0.38, respectively in sets of 14 improved varieties and 27 landraces of rice collected from different regions/zones of seven Indian states, which were lower to our result. Choudhury *et al*.^70^ reported a PIC value of 0.25 in 2630 Assam rice collections and PIC value of 0.23 in the whole northeast rice collection. These all findings indicated that the rice accession used in the present study material has larger genetic diversity. The marker RM 474 and RM 320 have higher discriminatory power to distinguish genotypes due to its high PIC value (0.747) and (0.749). The primer RM 87 and RM 480 showed lower PIC value (0.005) suggesting less discriminatory power of this primer under study.

Allelic richness (Ne) is a measure of genetic diversity and determines the flexibility of the population to adapt to various ecosystems^71^. In the present study, 65 polymorphic markers detected 147 alleles among 144 rice germplasm with an average of 2.26 alleles per locus. Similarly, Singh *et al*.^72^ also reported 112 alleles with an average of 3.11 alleles per locus in 729 varieties using 36 HvSSR markers. Anupam *et al*.^33^ also detected only 2–3 alleles in 74 rice germplasms comprised of indigenous landraces and improved variety and breeding lines of Tripura. Pauchauri *et al*.^69^ reported 2–4 alleles with an average of 2.7 alleles in a collection of landraces and improved variety. Islam *et al*.^73^ detected 2–3 alleles in the aromatic rice of Bangladesh. Whereas, Nachimuthu, *et al*.^74^ detected 2–7 alleles with an average of 3 alleles per locus in rice collected from India, South East Asia and America. Choudhary *et al*.^75^ reported a higher number of alleles (3.69) per locus with a range of 3 to 7 alleles in 100 major rice cultivars using 52 hyper-variable SSR markers. Das *et al*.^66^ also reported a higher number of alleles ranging from 2–11, with an average of 4.91 alleles per locus in Northeast rice germplasm. Edzesi *et al*.^76^ reported average 11.3 alleles per locus in China and Vietnam rice accession. Roy *et al*.^77^ also detected 2–21 alleles with an average of 8.49 alleles in rice accession comprised of Arunachal Pradesh landraces, basmati rice, local aromatic rice of Meghalaya, *aus* rice, *japonica* rice, *indica* rice. The discrepancy in the number of alleles detected might be due to the use of highly diverse genetic material and polymorphic DNA markers in their study.

Gene diversity similar to the present study has been reported by Chen *et al*.^78^. Ananadan *et al*.^40^ also reported 0.30 gene diversity in 426 ARC accessions, 25 tropical *japonica*, 57*indica* landraces, 127 breeding lines. Roy *et al*.^79^ reported 0.66 gene diversity in 26 rice accession of Arunachal Pradesh. Genotyping of 409 Asian rice accessions collected from 79 countries of the world using SSR markers revealed the genetic diversity of 0.68, which is higher than the present study observation^80^. Most of the diversity panel with global accessions have the gene diversity of 0.45 to 0.7^81,82^. These results on global accessions involving *indica, tropical japonica, temperate japonica* and wild relatives help to infer that, the diversity in a panel of 114 rice accession collected from North East India represents a considerable proportion of the genetic diversity that exists in the major rice-growing Asian continent. In the present study Nm value for some markers recorded more than 1, which is considered to be high as Nm value in self-pollinated crops lies below one^83^. The inclusion of sister lines or same landraces with different names or little amount of cross-pollination as farmers some time grows crops vary adjacent to each other might attributed to high Nm value.

Population structure analysis using model-based analysis is better than the frequentist approach of clustering since model-based clustering is based on Bayesian methods, in which certain parameters like correlated allele frequencies no-prior population information were used as defined in STRUCTURE software. Distance-based methods are usually easy to apply and are often visually appealing such as neighbor-joining to cluster multi-locus genotype data, but difficult to assess how confident one should be that the clusters obtained in this way are meaningful^84^.

The model-based Bayesian clustering algorithm approach assigns individuals to subpopulations. Among 114 genotypes 84 genotypes were pure and 30 rice accessions were admixture. The admixture was observed because of gene flow among genotypes through a small amount of natural cross-pollination as farmers grow rice crops adjacent to each other fields. The relatively small value of alpha (α = 0.1022) in the present study reveals that only a few individuals were admixed^72,85^. The assigning of genotypes to the subpopulation is based on the ancestry threshold of the individual. In the present study threshold of 80% was used for grouping which is more stringent and result in 30 genotypes as an admixture. A similar ancestral threshold of 80% to categorize an individual to a particular subpopulation was used by Salgotra *et al*.^59^, and Travis *et al*.^6^. However, if the ancestral threshold of 60% has been followed to categorize an individual according to Liakat Ali *et al*.^80^, then only 15 individuals have been categorized as an admixture.

In the present study, population structure analysis grouped 114 genotypes into three subpopulations named as P1, P2 and P3. Roy *et al*.^79^ also reported three number of a subpopulation in North East rice collection. Singh *et al*.^72^ reported three numbers of groups in Indian rice. Islam *et al*.^73^ reported three clusters in aromatic rice accessions. Das *et al*.^66^ reported four groups in a 91 rice landraces from Eastern and Northeastern India. Edzesi *et al*.^76^ reported seven subgroups in 628 rice accessions from China (507) and Vietnam (121) because of the big population and highly diverse population. Rathi *et al*.^86^ have reported ten subgroups in a population of 100 *indica* rice using 98 SSR markers. Anandan *et al*.^40^ grouped 96 rice accessions involving 70 ARC into two groups due to less number of genotypes and low diversity among individuals. In the present study grouping, pattern or number was different from other studies might be attributed to the use of different marker system and a different set of genotypes. In the present study, Banglami, ‘Rongadoria’ and ‘Kola Ahu’ was grouped in different subpopulations and ‘Inglongkiri’ and ‘Mazubiron’ was identified as admixture while Travis *et al*.^6^ reported ‘Banglami’, ‘BogaAhu’ and ‘Kola Ahu’ in Aus-1 and ‘Inglongkiri’ and ‘Mazubiron’ in Aus-2. Such differences might be attributed to use of fewer markers in the present study.

The P1 comprised of genotypes like ‘Banglami’, ‘Bizor’, ‘Bizor-2’, ‘Horin Kajuli’ and ‘Raja Ahu’ showing drought tolerance and high recovery rate. ‘Horin Kajuli’ was the best genotype of this group in terms of drought tolerance, root volume, fresh root weight and dry shoot weight. The best performing genotypes in P2 were ‘Kosamoni’ for drought tolerance and Hafa Ahu for root volume, fresh root weight and root length. The P3 consisted of 15 genotypes (Fig. 4), among which ‘Boga Gajeb’, ‘Gajef Sali-3’ and ‘Norin 18/Patnai 23’ showed moderate drought tolerance and rest genotypes were susceptible to drought. Among the genotypes of admixtured origin, 28% genotypes were drought-tolerant and others were identified as drought susceptible. For example, ‘Inglongkiri’, ‘ARC 10372’, ‘As 38/2’ showed drought tolerance. Among all 114 genotypes, Inglongkiri was the best genotypes for root volume, fresh root weight and dry shoot weight and root length. These indicated that different population identified using SSR markers also showed variation for different traits under study.

Results of AMOVA indicated that there was a higher proportion of variation among individuals and a lower proportion of variation among populations. Similar to this Jasim Aljumaili *et al*.^87^, Islam *et al*.^39^, Salgotra *et al*.^59^ and Singh *et al*.^72^ also reported a higher proportion of variation among individuals in the rice population. All the genotypes of the present study were collected from different regions of Assam covering lowland (Sali rice) and varied upland (Ahu rice) situation, which results in higher variation among individuals than among populations. Within individuals 6% variance was observed, it indicated the high purity of germplasm and has been maintained carefully without any mixture. A very high F_IT_ value has indicated a lack of heterozygosity most likely due to the inbreeding nature of rice (Nachimuthu *et al*.^74^). The F_ST_ inbreeding coefficient within subpopulations relative to the total provides a measure of the genetic differentiation between subpopulations^88^. The determination of F_ST_ using structure analysis for the subpopulation of the present study was 0.407 which indicated high differentiation between subpopulation because genotypes were collected from a wide range of ecology and topography. Wright^58^ proposed that values of F_ST_ 0.25 explain a very great differentiation between subpopulations; the range of 0.15 to 0.25 indicates moderate differentiation; while differentiation is not negligible if F_ST_ is 0.05 or less.

The PCoA analysis showed large genetic diversity and distinctness of populations, the first two principal coordinates explained 11.63 and 9.12% of the variance. A similar pattern of molecular variance was reported by Nachimuthu *et al*.^74^.

In all genotypic based clustering patterns of the present study, admixtures were distributed over subpopulation. Groupings of genotypes obtained through model-based analysis, unweighted neighbor-joining clustering and PCoA were incongruent to a large extent indicating real genetic differences among the genotypes under study at DNA level and perfectness of clustering of genotypes. Venn diagram analysis also showed more than 62% of co-linearity between rice germplasm grouping in neighbor-joining clustering and model-based population structure. A similar finding was also reported by Singh *et al*.^72^. Observed minor differences in a grouping of genotypes could be attributed to the difference in their methodology of grouping, as model-based analysis grouping is based on the Bayesian model approach whereas unweighted neighbor-joining clustering is distance-based approach in which genotypes with admixtures were not considered. The result of model-based analysis was more productive as it provides detail information about the genetic constitution of genotypes which help in the separation of admixture from pure genotypes. The result of Model-based analysis is robust for small population size^89^. Grouping or clustering of genotypes helps in identification of diverse parents to be used in the hybridization programme to create segregating progenies with maximum genetic variability for further selection^90^. Therefore the selection of genotypes from different population complimenting for different root traits and drought tolerance help in generating transgressive segregates for root traits and drought tolerance.

No correlation was observed between genotypic and phenotyping based clustering. which is obvious, because 61 SSR markers used in the present study were random SSR markers, not EST markers. SSR markers are present in both non-coding and coding region of genome^38,91,92^, but majorities of SSR markers are present in introns and might not affect the trait of interest directly^93^. Only a few of the SSR markers might be linked to genomic regions which influence the trait of interest. Phenotype is the resultant of genotype and environment effect. At molecular level phenotype is the outcome of many gene expression and interaction. Diversity based on phenotypic traits is influenced by environments^33–35^. Another reason could be that only a few markers may be linked with root, shoot and drought tolerance, therefore little variation is detected by the SSR markers in the present study. Farmers have selected land-races based on yield attributes and unknowingly they have selected for drought tolerance and better root system in upland rice germplasm of northeast India. Morphological traits were subjected to farmer’s selection pressure while SSR (DNA) markers were not the target of selection^94^. These reasons might leads to high chances of no correlation between genotypic and phenotypic based clustering. Silva *et al*.^95^ also reported a low correlation (0.35) between genotypic and phenotypic diversity in sorghum. Fufa *et al*.^96^ also observed a low correlation between genotypic based clustering and yield attributes based clustering in red winter wheat. Nazaphy *et al*.^97^ reported a low correlation of 0.049 between SSR marker-based clustering and phenotypic based clustering. According to Martinez *et al*.^98^ correspondence between molecular and phenotypic based diversity might be improved by analysing more numbers of morphological and DNA markers. Silva *et al*.^95^ stated that the poor correlation observed between the molecular and the phenotypic diversity matrices highlights the complementarity between the molecular and the phenotypic characterization to assist a breeding program. Hence, the combined use of phenotypic and molecular data is regarded as the best way to identify divergence among genotypes due to their complementary nature.

The present study revealed a high level of genetic diversity among the accession at DNA level, root phenotype and drought tolerance as the landraces of NE India are cultivated by farming community historically for many years in a diversified ecological niches such as near river basin, hills, hill slopes and plains, etc.

## Conclusion

In this present study, SSR based diversity analysis confirmed the existence of genetic diversity in a population of 114 rice genotypes. Based on various statistical methods, we identified three subpopulations along with 30 admixtures. These three subpopulations were highly differentiated from each other. The majority of variation was observed among individuals. The gene and allele based diversity analysis have indicated the existence of a broad genetic base in this population. The result of the model-based analysis is in close correspondence with the results of the neighbor-joining clustering and PCoA analysis. Thus, the results of this study indicate the scope for utilizing the genetic diversity results in association mapping analysis and selection of diversified genotypes for the development of variety from diversified groups complementing each other for various economical traits.

## References

[1] Food and Agriculture Organization of the United Nations. FAO Statistical Year Book - World Food and Agriculture (2012).

[2] FAO Rice Market Monitor, vol.XX(1), Rome, Itali (2017).

[3] Annonymous. Pocket Book of Agricultural Statistics 2018. Govt. Of India, New Delhi (2018).

[4] Fageria NK (2007). Yield physiology of rice. Journal of Plant Nutrition.

[5] Govt. of India [GOI], Ministry of Agriculture, (https://databank.nedfi.com/ content/land-use-4) (2015).

[6] Travis AJ (2015). Assessing the genetic diversity of rice originating from Bangladesh, Assam and West Bengal. Rice.

[7] Schatz MC (2014). Whole genome de novo assemblies of three divergent strains of rice, Oryza sativa, document novel gene space of aus and indica. Genome Biol..

[8] Civáň P, Craig H, Cox CJ, Brown TA (2015). Three geographically separate domestications of Asian rice. Nat Plants.

[9] Kim, H. *et al*. Population dynamics among six major groups of the Oryza rufipogon species complex, wild relative of cultivated Asian rice. *Rice* **9**(1) (2016). 10.1186/s12284-016-0119-0 PMC5059230 27730519

[10] Londo JP (2006). Phylogeography of Asian wild rice, Oryza rufipogon, reveals multiple independent domestications of cultivated rice, Oryza sativa. Proc. Nat. Acad. Sci. USA.

[11] Khush GS (1997). Origin, dispersal, cultivation and variation of rice. Plant Mol. Biol..

[12] Bin Rahman ANMR, Zhang J (2016). Flood and drought tolerance in rice: opposite but may coexist. Food and Energy Security.

[13] Gamuyao R (2012). The protein kinase pstol1 from traditional rice confers tolerance of phosphorus deficiency. Nature.

[14] Xu K (2006). Sub1A is an ethylene-response-factor-like gene that confers submergence tolerance to rice. Nature.

[15] Xu K, Mackill DJ (1996). A major locus for submergence tolerance mapped on rice chromosome 9. Molecular Breeding.

[16] Henry A (2011). Variation in root system architecture and drought response in rice (Oryza sativa): Phenotyping of the Oryza SNP panel in rainfed lowland fields. Field Crops Research.

[17] Gowda VRP (2012). water uptake dynamics under progressive drought stress in diverse accessions of the Oryza SNP panel of rice (Oryza sativa). Functional Plant Biology.

[18] Jagadish SV (2010). Physiological and proteomic approaches to address heat tolerance during anthesis in rice (Oryza sativa L.). J. Exp. Bot..

[19] Vikram P, Singh AK, Singh SP (2010). Sequence analysis of Nagina-22 drought tolerant ESTs for drought specific SSRs. Int. J. Plant Sci..

[20] Ye C (2015). Identifying and confirming quantitative trait loci associated with heat tolerance at the flowering stage in different rice populations. BMC Genet..

[21] Mutum RD (2016). Identification of novel miRNAs from drought tolerant rice variety. Scientific Reports.

[22] Yu Y (2017). The complete chloroplast genome sequence of Oryza sativa aus-type variety Nagina-22 (Poaceae). Mitochondrial DNA Part B: Resources.

[23] Kilasi, N. L. *et al*. Heat stress tolerance in rice (Oryza sativa L.): Identification of Quantitative Trait Loci and Candidate Genes for Seedling Growth Under Heat Stress. *Frontiers in Plant Science* **9**, 1–11, https://www.frontiersin.org/article/, 10.3389/fpls.2018.01578/full (2018). 10.3389/fpls.2018.01578 PMC6221968 30443261

[24] Chen S (2011). Genetic analysis and molecular mapping of a novel recessive gene Xa34(t) for resistance against Xanthomonas Oryzae Pv. Oryzae. Theoretical and Applied Genetics.

[25] Torres RO, McNally KL, Cruz CV, Serraj R, Henry A (2013). Screening of rice Genebank germplasm for yield and selection of new drought tolerance donors. Field Crops Res..

[26] Uga Y, Kitomi Y, Ishikawa S, Yano M (2015). Genetic improvement for root growth angle to enhance crop production. Breeding Science.

[27] Comas LH (2013). Root traits contributing to plant productivity under drought. Frontiers in Plant Science.

[28] Vadez V, Rao JS, Mathur PB, Sharma KK (2013). DREB1A promotes root development in deep soil layers and increases water extraction under water stress in groundnut. Plant Biology.

[29] Wasaya A, Zhang X, Fang Q, Yan Z (2018). Root phenotyping for drought tolerance: A review. Agronomy.

[30] Gowda VRP (2011). Root biology and genetic improvement for drought avoidance in rice. Field Crops Research.

[31] Uga Y (2013). Control of Root System Architecture by DEEPER ROOTING 1 Increases Rice Yield under Drought Conditions. Nature Genetics.

[32] Ingram, K.T., Bueno, F. D., Namuco, O. S., Yambao, E. B. & Beyrouty, C. A. Rice root traits for drought resistance and their genetic variation. IRRI, Philippines. In Kirk, G. J. D. ed., Rice Roots. Nutrient and Water Use 67–77 (1994).

[33] Anupam (2017). Genetic diversity analysis of rice germplasm in Tripura state of northeast India using drought and blast linked markers. Rice. Science.

[34] Shehzad T, Okuizumi H, Kawase M, Okuno K (2009). Development of SSR-based sorghum (Sorghum bicolor (L.) Moench) diversity research set of germplasm and its evaluation by morphological traits. Genetic Resources and Crop Evolution.

[35] Last L, Lüscher G, Widmer F, Boller B, Kölliker R (2014). Indicators for genetic and phenotypic diversity of Dactylis glomerata in Swiss permanent grassland. Ecological Indicators.

[36] McCouch SR (1997). Microsatellite marker development, mapping and applications in rice genetics and breeding. Plant molecular biology..

[37] Gupta PK, Varshney RK (2000). The development and use of microsatellite markers for genetic analysis and plant breeding with emphasis on bread wheat. Euphytica.

[38] Vieira MLC (2016). Microsatellite Markers: What They Mean and Why They Are so Useful. Genetics and Molecular Biology.

[39] Islam MZ (2018). Diversity and population structure of red rice germplasm in Bangladesh. PLoS ONE..

[40] Anandan, A., Anumalla, M., Pradhan, S. K. & Ali, J. Population structure, diversity and trait association analysis in rice (Oryza sativa L.) germplasm for early seedling vigor (ESV) using trait linked SSR markers. *PLoS ONE* **11**(3) (2016). 10.1371/journal.pone.0152406 PMC4816567 27031620

[41] Uga Y, Okuno K, Yano M (2011). Dro1, a major QTL involved in deep rooting of rice under upland field conditions. J. Expt. Bot..

[42] Shashidhar, H. E.; Henry, A. & Hardy, B. Methodologies for root drought studies in rice. *Los Banos:International Rice Research Institute* (2012).

[43] Swain P, Anumalla M, Prusty S, Marndi BC, Rao GJN (2014). Characterization of some Indian native landrace rice accessions for drought tolerance at seedling stage. Australian J. Crop Sci..

[44] IRRI, Standard Evaluation System for Rice. International Rice Research Institute, Manila (2002).

[45] Reynolds SG (1970). The gravimetric method of soil moisture determination, Part 1 A study of equipments and methodological problems. Journal of hydrolog.

[46] Plaschke J, Ganal MW, Röder MS (1995). Detection of genetic diversity in closely related bread wheat using microsatellite markers. Theor. Appl. Genet..

[47] Panaud O, Chen X, McCouch SR (1996). Frequency of microsatellite sequences in rice (Oryza sativa L.). Genome.

[48] Temnykh S (2000). Mapping and genome organization of microsatellite sequences in rice (Oryza sativa L.). Theor Appl Genet..

[49] R Core Team R: A language and environment for statistical computing. R Foundation for Statistical Computing, Vienna, Austria. URL, http://www.R-project.org/ (2013).

[50] Pritchard J, Stephens M, Donnelly P (2000). Inference of population structure using multilocus genotype data. Genetics.

[51] Evanno G, Regnaut S, Goudet J (2005). Detecting the number of clusters of individuals using the software STRUCTURE: a simulation study. Mol. Ecol..

[52] Earl DA, VonHoldt BM (2012). Structure Harvester: A website and program for visualizing STRUCTURE output and implementing the Evanno method. Conserv Genet Resour..

[53] Peakall R, Smouse PE (2012). GenAlEx 6.5: genetic analysis in Excel. Population genetic software for teaching and research-an update. Bioinformatics.

[54] Botstein D, White RL, Skolnick M, Davis RW (1980). “Botstein.” Am J Hum. Gen..

[55] Perrier, X. & Jacquemoud-Collet, J. P. DARwin software Version 5.0.155. CIRAD, http://darwin.cirad.fr/darwin (2006).

[56] Oliveros, J. C. V. An interactive tool for comparing lists with Venn diagrams. BioinfoGP, CNB-CSIC Key: citeulike, 6994833 (2007).

[57] Mantel N (1967). The detection of disease clustering and a generalized regression approach. Cancer Research.

[58] Wright, S. Evolution and the Genetics of Populations, Vol. 4. University of Chicago Press, Chicago (1978).

[59] Salgotra RK (2015). Genetic Diversity and Population Structure of Basmati Rice (Oryza sativa L.) Germplasm Collected from North Western Himalayas Using Trait Linked SSR Markers. PLoS ONE.

[60] Sandhu N, Kumar A (2017). Bridging the Rice Yield Gaps under Drought: QTLs, Genes, and Their Use in Breeding Programs.”. Agronomy.

[61] Bhandari, H. R. *et al*. Assessment of genetic diversity in crop plants - an overview. *Advances in Plants & Agriculture Research* **7**(3) (2017).

[62] Swingland, I. R. Biodiversity, Definition of. Encyclopedia of Biodiversity 1, 377–390 (2001).

[63] Idrees M, Irshad M (2014). Molecular markers in plants for analysis of genetic diversity: a review. Eur. Acad. Res..

[64] Kesawat SM, Das BK (2009). Molecular markers: Its application in crop improvement types of molecular markers. J. Crop Sci. Biotech..

[65] Hildebrand E, Torney DC, Wagner RP (1992). Informativeness of polymorphic DNA markers. Los Alamos Science.

[66] Das B (2013). Genetic diversity and population structure of rice landraces from eastern and north-eastern states of India. BMC Genetics..

[67] Behera L (2012). Assessment of genetic diversity in medicinal rices using microsatellite markers. Australian Journal of Crop Science.

[68] Shah, S. M., Naveed, S. A., & Arif, M. Genetic diversity in basmati and non-basmati rice varieties based on microsatellite markers. *Pakistan Journal of Botany, 45(SPL.ISS)*, 423–431 (2013).

[69] Pachauri V (2013). Molecular and Morphological Characterization of Indian Farmers Rice Varieties (Oryza sativa L.). Australian Journal of Crop Science..

[70] Choudhury DR (2014). Analysis of genetic diversity and population structure of rice germplasm from north-eastern region of India and development of a core germplasm set. PLoS ONE.

[71] Greenbaum G (2014). Allelic richness following population founding events - A stochastic modeling framework incorporating gene flow and genetic drift. PLoS ONE.

[72] Singh N (2016). Genetic diversity trend in Indian rice varieties: An analysis using SSR markers. BMC Genetics.

[73] Islam, M. Z. *et al*. Variability assessment of aromatic rice germplasm by pheno-genomic traits and population structure analysis, Scientific Reports volume 8, Article number: 9911 (2018). 10.1038/s41598-018-28001-z PMC6028394 29967407

[74] Nachimuthu, V. V. *et al*. Analysis of population structure and genetic diversity in rice germplasm using SSR markers: An initiative towards association mapping of agronomic traits in Oryza sativa. *Rice* **8**(1) (2015). 10.1186/s12284-015-0062-5 PMC4583558 26407693

[75] Choudhary Gangaprasad, Ranjitkumar Nagireddy, Surapaneni Malathi, Deborah Dondapati Annekitty, Vipparla Abhilash, Anuradha Ghanta, Siddiq Ebrahimali Abubacker, Vemireddy Lakshminarayana Reddy (2013). Molecular Genetic Diversity of Major Indian Rice Cultivars over Decadal Periods. PLoS ONE.

[76] Edzesi WM (2016). Genetic diversity and elite allele mining for grain traits in rice (Oryza sativa l.) by association mapping. Frontiers in Plant Science.

[77] Roy S (2016). Genetic diversity and structure in hill rice (Oryza sativa L.) landraces from the North Eastern Himalayas of India. BMC Genetics.

[78] Chen H (2011). Development and application of a set of breeder-friendly SNP markers for genetic analyses and molecular breeding of rice (Oryza sativa L.). Theoretical and Applied Genetics.

[79] Roy S (2015). Genetic diversity and population structure in aromatic and quality rice (Oryza sativa L.) landraces from north-eastern India. PLoS ONE.

[80] Liakat AM (2011). A Rice Diversity panel evaluated for genetic and agro-morphological diversity between subpopulations and its geographic distribution. Crop Science..

[81] Garris AJ (2005). Genetic structure and diversity in Oryza sativa L. Genetics.

[82] Ni J, Colowit PM, Mackill DJ (2002). Evaluation of genetic diversity in rice subspecies using microsatellite markers. Crop Sci..

[83] Govindaraju DR (1989). Variation in gene flow levels among predominantly self‐pollinated plants. Journal of Evolutionary Biology.

[84] Bowcock AM (1994). High resolution of human evolutionary trees with polymorphic microsatellites. Nature.

[85] Hurtado, L. P. *et al*. An overview of STRUCTURE: Applications, parameter settings, and supporting software. *Frontiers in Genetics* 4(MAY), 1–13 (2013). 10.3389/fgene.2013.00098 PMC3665925 23755071

[86] Rathi S (2014). Association studies of dormancy and cooking quality traits in direct-seeded indica rice. Journal of Genetics.

[87] Aljumaili, J. *et al*. Genetic diversity of aromatic rice germplasm revealed by SSR markers. *BioMed Res Int*. 1–11 (2018). 10.1155/2018/7658032 PMC5874984 29736396

[88] Ochoa & Storey, FST and kinship for arbitrary population structures I: Generalized definitions (2016). 10.1371/journal.pgen.1009241 PMC7846127 33465078

[89] Yamasaki M, Ideta O (2013). Population structure in Japanese rice population. Breed Sci..

[90] Barrett BA, Kidwell KK (1998). AFLP-based genetic diversity assessment among wheat cultivars from the Pacific Northwest. Crop Sci..

[91] Pérez-Jiménez M, Besnard G, Dorado G, Hernandez P (2013). Varietal tracing of virgin olive oils based on plastid DNA variation profiling. PLoS One.

[92] Phumichai C, Phumichai T, Wongkaew A (2015). Novel chloroplast microsatellite (cpSSR) markers for genetic diversity assessment of cultivated and wild Hevea rubber. Plant Mol Biol Report.

[93] Temnykh S (2001). Computational and experimental analysis of microsatellites in rice (Oryza sativa l.): frequency, length variation, transposon associations, and genetic marker potential. Genome Research.

[94] Casa A (2005). Diversity and selection in sorghum: simultaneous analyses using simple sequence repeats. Theor Appl Genet..

[95] da Silva M. J. *et al*. Phenotypic and molecular characterization of sweet sorghum accessions for bioenergy production. *PLoS ONE* **12**(8) (2017). 10.1371/journal.pone.0183504 PMC5560702 28817696

[96] Fufa H (2005). Comparison of phenotypic and molecular marker-based classifications of hard red winter wheat cultivars. Euphytica.

[97] Najaphy A, Parchin RA, Farshadfar E (2012). Comparison of phenotypic and molecular characterizations of some important wheat cultivars and advanced breeding lines. AJCS..

[98] Martinez L, Cavagnaro P, Masuelli R (2005). Evaluation of diversity among Argentine grapevine (Vitis vinifera L.) varieties using morphological data and AFLP markers. Elect. J Biotech.

